# Dynamic self-guiding analysis of Alzheimer's disease

**DOI:** 10.18632/oncotarget.4221

**Published:** 2015-05-20

**Authors:** Alexei Kurakin, Dale E. Bredesen

**Affiliations:** ^1^ Mary S. Easton Center for Alzheimer's Disease Research, Department of Neurology, University of California, Los Angeles, CA, USA; ^2^ Buck Institute for Research on Aging, Novato, CA, USA

**Keywords:** Alzheimer's disease, cancer, degeneration, cytoskeletal networks, cell adhesions

## Abstract

We applied a self-guiding evolutionary algorithm to initiate the synthesis of the Alzheimer's disease-related data and literature. A protein interaction network associated with amyloid-beta precursor protein (APP) and a seed model that treats Alzheimer's disease as progressive dysregulation of APP-associated signaling were used as dynamic “guides” and structural “filters” in the recursive search, analysis, and assimilation of data to drive the evolution of the seed model in size, detail, and complexity. Analysis of data and literature across sub-disciplines and system-scale discovery platforms suggests a key role of dynamic cytoskeletal connectivity in the stability, plasticity, and performance of multicellular networks and architectures. Chronic impairment and/or dysregulation of cell adhesions/synapses, cytoskeletal networks, and/or reversible epithelial-to-mesenchymal-like transitions, which enable and mediate the stable and coherent yet dynamic and reconfigurable multicellular architectures, may lead to the emergence and persistence of the disordered, wound-like pockets/microenvironments of chronically disconnected cells. Such wound-like microenvironments support and are supported by pro-inflammatory, pro-secretion, de-differentiated cellular phenotypes with altered metabolism and signaling. The co-evolution of wound-like microenvironments and their inhabitants may lead to the selection and stabilization of degenerated cellular phenotypes, via acquisition of epigenetic modifications and mutations, which eventually result in degenerative disorders such as cancer and Alzheimer's disease.

## INTRODUCTION

Due to the introduction and success of high-throughput discovery platforms and the ever-increasing productivity of experimental research, the rate of data generation noticeably exceeds the rate at which data are translated into answers, solutions, products, and therapies. It is possible therefore that many answers and solutions are already present in research literature and databases but in a disarranged and dispersed form, similar to the images hidden in the pieces of jigsaw puzzles that accumulate in a growing number of separate boxes, in which they are neatly sorted on the basis of their size, color, shape, or weight. After all, all sciences and disciplines study the same reality but focus narrowly and intensely on its different aspects, which they often interrogate and describe in a highly idiosyncratic manner.

To illustrate the feasibility of synthesizing knowledge across sub-disciplines and high-throughput discovery platforms we applied an evolutionary algorithm to analyze the research literature and data pertaining to Alzheimer's disease (AD) and cellular signaling and protein interaction networks. Briefly, the algorithm starts with “bringing forward” from the “background” of research data a small set of essential concepts and their relationships, a core model, in this case a model of Alzheimer's disease. The model is given a large experimental dataset generated by system-scale technologies, which is deemed to be of utmost importance, according to the model. In this particular case, it is a protein interaction network centered on amyloid-beta precursor protein (APP), by common consensus one of the key players in the pathogenesis of Alzheimer's disease. Both the model and the accompanying dataset are open and dynamic in the sense that any element or relationship can be added or discarded by “bringing it forward” from or “sending it back” into “background”, without imposing a judgment as to what is “right” or what is “wrong”. In the first step, the seed model is used as a guide for identifying and analyzing the meta-reviews and reviews that seem to be most pertinent according to the model. In the course of literature analysis, the concepts and their interrelations that contain highest densities of dataset elements (e.g., names of the proteins physically and functionally associated with APP) are “brought forward” from “background” into the model, thus shaping, refining, and enlarging the model. Next, the refined and enlarged model is used as a guide for identifying and analyzing more detailed information contained in the reviews, topical reviews, and original research articles that are deemed to be most pertinent according to the refined model. The procedure is repeated in a recursive manner, allowing the model to evolve and to grow in size, detail, and complexity by assimilating and structuring both the experimental dataset and the relevant data accumulating in research databases. Because the model and its accompanying dataset are open and dynamic, and the search and analysis are not exhaustive and comprehensive, the “validity” of an evolving model is equated to its practical worth, judged by the increasing ability of an evolving model to make increasingly specific and accurate predictions about experimental reality, thus increasing the rate and/or extent of data assimilation, while minimizing inconsistencies. As demonstrated below, in many cases, the experimental data needed to verify model predictions are already present in research databases.

The overall procedure is reminiscent of the progressive forming of a gestalt figure/ground, achieved by differentiating and accentuating a growing number of progressively finer elements in the “background”, bringing them “forward” and structuring them into an increasingly clear, detailed, and coherent figure/image. The process is inherently dynamic and what is included in figure and what in ground is subject to change and evolution. Provided that the unwarranted fixation on a particular figure/model is avoided and the relation between figure and ground is maintained free-flowing and dynamic, the process remain plastic, allowing for “gestalt switches” and alternative interpretations of the same data. Moreover, the rich and complex tapestry of “background”, i.e., the knowledge and data accumulating in research databases, becomes an inexhaustible source of alternative and complementary gestalts-models that can be readily generated via dynamic structuring, de-structuring, and recombination of concepts and data. The generated models-gestalts can be made to grow, evolve, and integrate into larger-scale models-gestalts by making them to compete and cooperate in the assimilation, integration, and structuring of large datasets generated by high-throughput, system-scale technologies. Integration and synthesis of knowledge by recursive assimilation (ISKRA) may potentially form a conceptual basis of a new information technology that may help to accelerate the translation of accumulating data and knowledge into practical solutions, products, and therapies.

## RESULTS

### Cell metabolism, signaling, and organizational plasticity

A living cell can operate in a number of alternative metabolic modes, switching from one mode to another. The recent discovery of the so-called metabolic cycle revealed that alternative metabolic modes are closely associated with mode-specific gene expression and intracellular organization, as reflected in mode-specific cytoskeletal and membrane organization/dynamics, all coordinated and controlled via cell signaling [13-15].

Simplifying, one can discriminate between two mechanisms by which cells satisfy their energy needs – oxidative phosphorylation and “(glyco)lytic” modes of metabolism (broadly defined). The latter includes anaerobic processes that do not require oxygen, e.g., fermentation (useful in hypoxic conditions), and aerobic mechanisms, such as aerobic glycolysis. As first pointed out by Warburg, oxidative phosphorylation is an evolutionarily more recent, advanced, and efficient mechanism of energy generation. However, it requires a high degree/extent of intracellular organization, ordering, and coherence; in particular, well performing, coupled, and coordinated cytoskeletal, mitochondrial, and endoplasmic reticulum (ER) networks. A high degree/extent of intracellular order and coherence requires and is required for oxidative phosphorylation, i.e., they are interdependent. One cannot exist without the other, because they enable and sustain each other [16]. The “(glyco)lytic” type of energy production is a set of more primitive and ancient mechanisms. Although less efficient as compared to oxidative phosphorylation, these mechanisms are more flexible, locally autonomous, and do not require a high degree/extent of intracellular organization and coherence, allowing for fast and flexible adaptations (local and global) in rapidly changing, unpredictable, and/or hostile/offensive environments. Stressed and adapting cells often switch to more flexible, “(glyco)lytic” modes of energy generation because such modes of functioning increase their chances for survival and successful adaptation in stressful and rapidly changing environments. In fact, proliferating cells seem to switch to “(glyco)lytic” modes of metabolism during each cell division because they need to dismantle, to renew/duplicate, and to reassemble their cytoskeleton, mitochondrial network, ER, and other organelles each time they divide. Therefore, in addition to stress, “(glyco)lytic” modes of metabolism are also associated with rapid cell proliferation [17, 18].

Epithelial-to-mesenchymal transition (EMT) is a fundamental process that endows polarized epithelial cells with the plasticity required during developmental morphogenesis and organogenesis. Less drastic changes in epithelial plasticity involve the transient acquisition of a migratory phenotype, accompanied by loss of cell polarity without sustained changes in gene expression. This phenotype applies to growth-factor-induced “scattering” of cultured epithelial cell, tubulogenesis and branching in the mammary gland, and tissue remodeling in wound healing [19]. The plasticity of epithelial cells allows them to go through multiple rounds of EMT and its reversal, MET (mesenchymal-to-epithelial transition). In all tissue contexts, key events accompanying EMT involve dissolution of cell-cell adhesions and reorganization of cytoskeletal architecture and, in many cases, acquisition of the ability to migrate and remodel extracellular matrix (ECM) [19-21].

**Figure 1:**
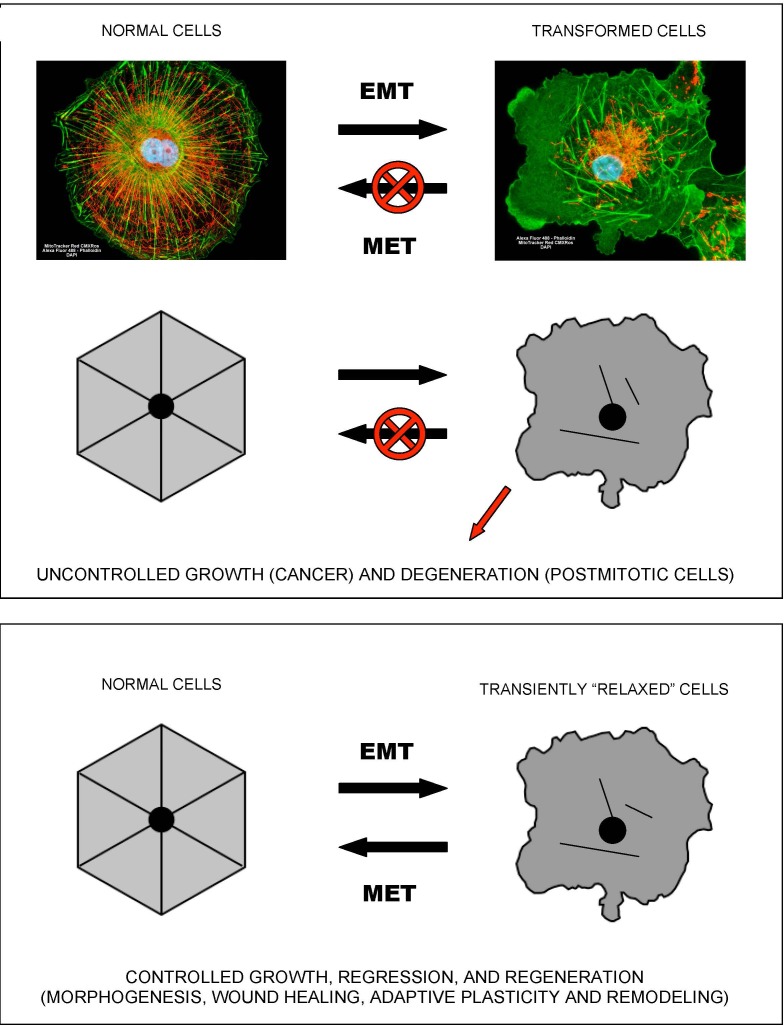
Epithelial-to-mesenchymal-like organizational transitions The shown schematics and cell images illustrate the concept of EMT-like, organizational transitions, which involve coordinated switches in cell adhesions, cytoskeletal organization/dynamics, metabolism, and signaling. The cell image shown on the left is a normal kidney fibroblast in cell culture (CV-1 cells), whereas the cell image on the right is its SV40-transformed derivative (COS-7 cells). Note the dramatic structural collapse of the mitochondrial and actin filamentous networks in transformed cells (F-actin – GREEN; mitochondria – RED). Images courtesy of Carl Zeiss Microscopy GmbH.

In terms of cellular signaling, studies in the fields of cancer and inflammation suggest that NF-kB and SirT1 activities are causatively or accidentally associated with two alternative modes of cellular functioning [22]. NF-kB signaling is associated with pro-migratory, pro-secretion, pro-inflammatory phenotypes and “(glyco)lytic” modes of metabolism. At the sub-cellular level, this type of metabolism is locally autonomous and thus does not require a high degree of intracellular coordination and long-range coherence. Correspondingly, the “NF-kB” mode of cell functioning is associated with a “fluid” form of intracellular organization, which characterizes mobile cells, such as macrophages, “activated” cells within wounds (which undergo a reversible EMT-like transition to become active and mobile), and cancer cells (Figure 1). Such cells typically exhibit proliferative, migratory, pro-secretion, pro-inflammatory phenotypes, and many of them are in fact attracted to the sources of and/or environments with high levels of reactive oxygen species, such as wounds. As an example, leukocytes locate and navigate wounds by migrating down tissue-scale, concentration gradients of hydrogen peroxide, a long-lived reactive oxygen species that is generated in wounds and acts as a chemoattractant for white blood cells [23].

**Figure 2:**
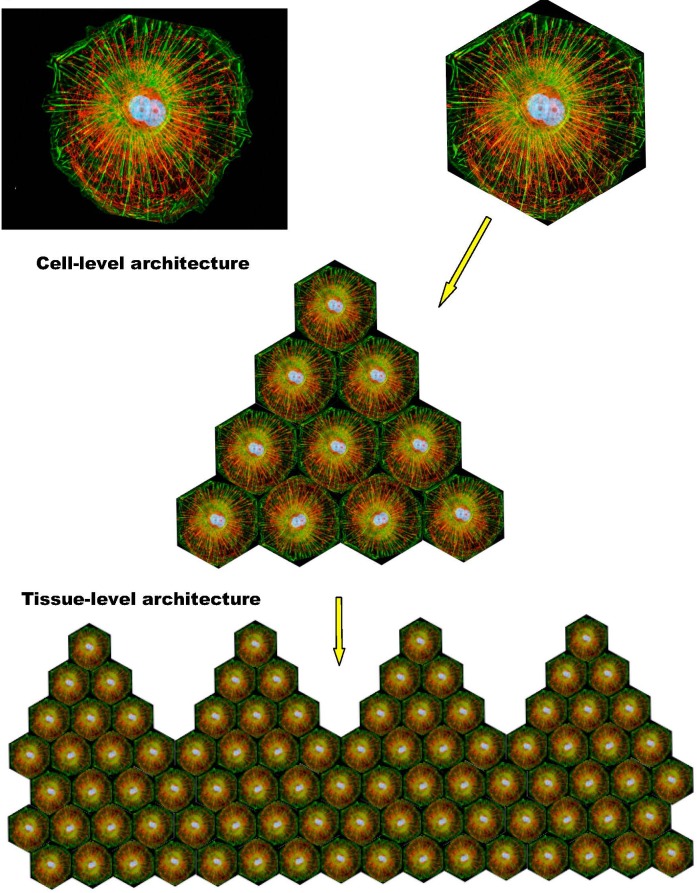
Integration of individual cytoskeletal networks into multicellular architectures The shown schematics illustrate the interrelations between cell- and tissue-level architectures. Dynamic coupling of dynamic cytoskeletal networks via dynamic cell adhesions enables the emergence, persistence, and prosperity of reconfigurable and adaptable, multi-scale, multicellular architectures/networks.

**Figure 3:**
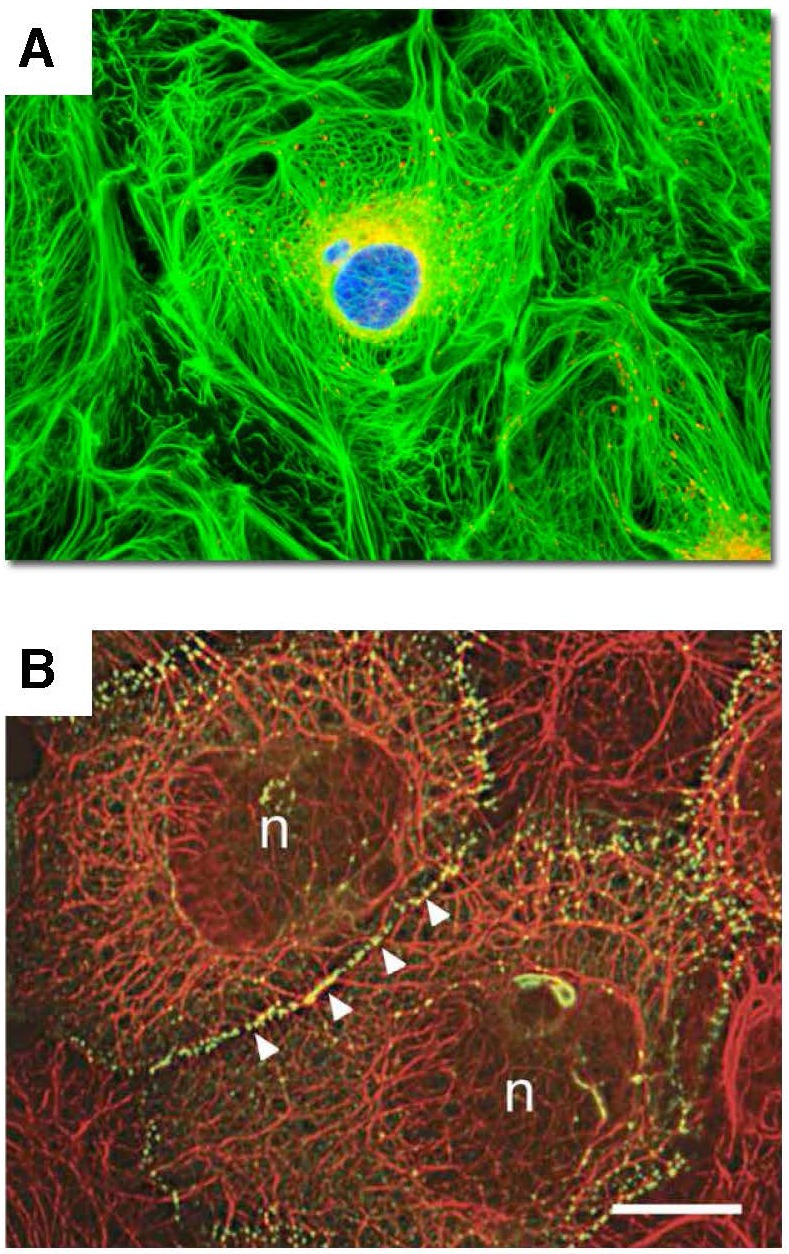
Integration of keratin cytoskeletal networks in cultured cells Cell images illustrate the integration of individual intermediate filament networks via dynamic cell adhesions into large-scale, collective, cytoskeletal architectures. **A.** Rat kangaroo kidney epithelial cells in culture. Keratin – GREEN; mitochondria – RED. Image courtesy of Olympus America; **B.** Skin epidermal keratinocytes in culture. Keratin – RED; cell adhesions – GREEN; (n) nucleus. Reproduced from Kim S. and Coulombe P.A. [81]. Courtesy of Dr. Kathleen Green.

SirT1 signaling is associated with resolution of inflammation and a switch to a more efficient mode of metabolism. However, this type of metabolism apparently requires a high degree of intracellular coordination and long-range coherence, and, correspondingly, a relatively “rigid” and stable intracellular organization supported by appropriate cytoskeletal and membrane structures and dynamics. In addition, it also requires intact and properly functioning cell-to-cell and/or cell-to-matrix adhesions. Both “rigid” intracellular organization and stable adhesion/coupling are necessary pre-requisites for the emergence and stability of tissue-level organization/architecture (e.g., epithelial sheets). Once emerged, a structured and coherent organization of interconnected cells and ECM behaves as a multicellular whole, which supports individual cells and is supported by individual cells, in a mutually reinforcing manner. Stable coupling of stable cellular cytoskeletons via stable cell adhesions is required for efficient cell-cell communications and the long-range coordination and coherence of individual activities, which define the structure, function, and performance of the multicellular whole (Figures 2, 3).

**Figure 4:**
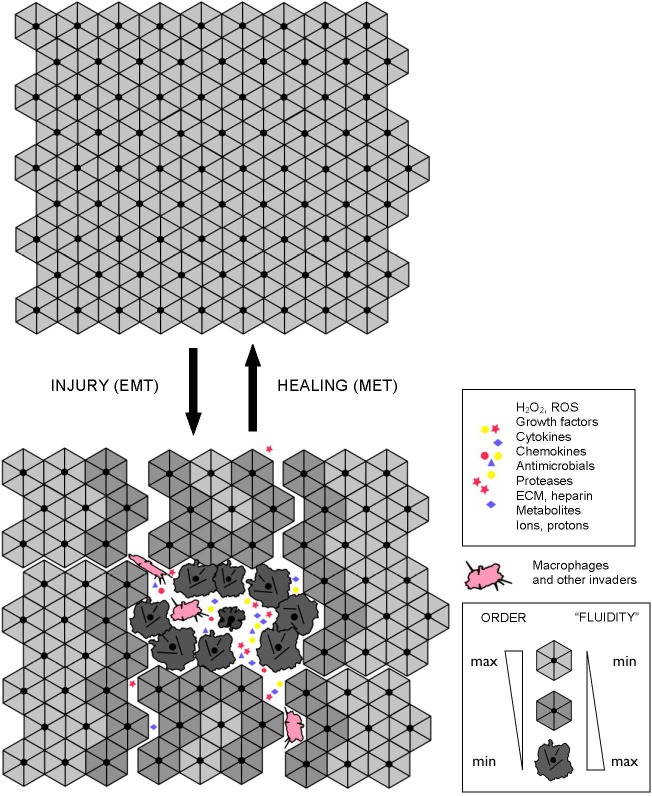
Interdependent molecular-, cell-, and tissue-level dynamics in wound repair and regeneration The shown schematics illustrate reversible, EMT-like, structural/metabolic/signaling transitions that enable wound repair, regeneration, and adaptability of multicellular architectures. The success or failure of tissue repair, adaptation, and plasticity depends on the reversible dynamics that take place at the molecular-, cell-, and tissue levels of organization in an interdependent manner.

Tissue malformations, recognized by pathologists as dysplasias and anaplasias, indicate a localized breakdown or “melting” of collective cellular organization and, by implication, a breakdown or “melting” of coupled cytoskeletons and cell adhesions on which tissue connectivity, structure, and performance rely. Destabilization of cellular cytoskeletons and/or adhesions, whether regulated (e.g., due to signaling) or unintended (e.g., due to stress, wounding, or hypoxia), can cause a reversible, EMT-like transition to an alternative mode of cellular organization and functioning, which allows individual cells to survive and act independently of the disrupted or destabilized collective organization (Figure 4). Such a switch leads to cell “individualization”, accompanied by cell de-differentiation and a conversion to a more primitive cellular phenotype, such as mesenchymal or amoeboid phenotypes. When stabilized and supported by appropriate changes in metabolism and gene expression, a “fluid”, individualistic mode may persist indefinitely. It is worth noting that, although the adjectives “rigid” and “fluid” are relatively accurate and convenient metaphors, they should not be taken literally. Increased “fluidity” refers to the enhanced pliability of cellular organization and dynamics, which may have different causes, such as, for example, a shift to a different type of cytoskeletal organization/dynamics and/or metabolism, the acceleration of turnover rates in steady-state structural dynamics, and/or an increased frequency of dynamic instabilities in intracellular organization.

Shifting a balance from “rigid”/collective modes to increasingly “fluid”/individualistic modes of cellular organization and functioning (or switching to and irreversibly locking in a “fluid”/individualistic mode) may lead to large-scale tissue malformations and/or uncontrolled cell proliferation, manifested as hyperplasias and neoplasias. Consequently, in the course of cancer progression to metastasis, cancer cells commonly acquire mutations targeting cell adhesions and undergo EMT, stabilizing ensuing de-differentiated phenotypes by altering their metabolism, signaling, and gene expression. As an example, E-cadherin, a classical cell-cell adhesion receptor and a key regulator of tissue architecture, is a tumor suppressor that mediates contact inhibition of cell proliferation and inhibits invasion and metastasis in a variety of contexts. E-cadherin function is often lost during progression of cancer to metastasis. It is worth noting that presenilin 1 (PS1), a catalytic component of the gamma-secretase complex, and one of the three proteins mutated in the early-onset familial Alzheimer's disease (FAD), colocalizes with cadherins upon formation of cell-to-cell contacts and stabilizes cadherin-based adhesions. Upon conditions that promote cell-cell dissociation, including calcium influx and apoptosis, E-cadherin is processed by a PS1/gamma-secretase activity. This cleavage promotes a disassembly of adherens junctions and leads to a release of a cytoplasmic E-cadherin fragment that translocates to the nucleus and triggers transcriptional responses [24, 25]. Pertinently, the loss of E-cadherin and E-cadherin-mediated cell adhesions activates NF-kB signaling, which presumably leads to changes in metabolism and gene expression [26, 27].

YAP1 and TAZ, two homologous transcriptional co-activators, are key effectors of the highly conserved Hippo pathway that relays signals from the plasma membrane (e.g., from adhesion receptors) into the nucleus to regulate transcriptional programs responsible for cell proliferation, survival, and differentiation. Activated YAP1/TAZ relocate to the nucleus and promote EMT and adhesion-independent growth in a number of contexts, including the loss of E-cadherin and E-cadherin-mediated adhesions [28]. In addition, the Hippo pathway and YAP1 were recently identified as effectors of E-cadherin-mediated contact inhibition of cell proliferation [29]. YAP1 and TAZ are hyperactivated in many cancers, and therefore considered as promising anti-cancer drug targets. However, YAP1/TAZ are also considered as promising targets for regenerative medicine, because transient activation of YAP1/TAZ is required for regenerative de-differentiation and proliferation in the context of tissue injury, promoting tissue repair and regeneration [28, 30] (Figure 4).

The processing and signaling of amyloid-beta precursor protein (APP) are remarkably similar to that of E-cadherin and its neuronal counterpart N-cadherin. Specifically, members of the ADAM (“a disintegrin-and-metalloprotease”) family, in particular ADAM10, also known as “alpha-secretase” in the context of APP processing, are principal proteases that process E-cadherin, N-cadherin, and APP to release their extracellular domains. Ectodomain shedding of N-cadherin by ADAM10 occurs in response to calcium influx, followed by the subsequent PS1/gamma-secretase-mediated cleavage of N-cadherin to yield a N-cadherin C-terminal fragment, N-Cad/CTF2, which functions as a potent repressor of CBP/CREB-mediated transcription [24, 31, 32]. APP and E-cadherin are processed in a similar manner by the same proteases to yield C-terminal fragments that translocate to the cell nucleus and trigger transcriptional responses [24, 33]. Moreover, YAP1 and TAZ, downstream effectors of E-cadherin signaling, were recently identified as effectors of APP signaling, whose transcriptional activation was dependent on APP processing by the gamma-secretase complex [12]. Altogether, APP processing and signaling in synapses (specialized neuronal cell-cell adhesions) appear to proceed via evolutionarily conserved pathways that are shared between epithelial adhesions and neuronal adhesions/synapses, including the Hippo signaling pathway and its effectors YAP1 and TAZ that control EMT.

It is worth noting that the formation of coherent multicellular assemblies, which proceeds via establishing cell-cell adhesions and cell differentiation, and the disbanding of multicellular assemblies, which proceeds via dissolution of cell adhesions and cell de-differentiation, are apparently mediated by ancient and highly conserved molecular mechanisms. For example, a number of unicellular eukaryotes, such as *Dictyostelium discoideum* (slime mold) and choanoflagellates, live as solitary individuals. However, in response to diverse environmental cues, in particular, starvation, they acquire polarized phenotype, differentiate, form cell-cell junctions, deposit shared ECM, and survive as a coherent colony, a primitive multicellular organism. Genome sequencing efforts unexpectedly revealed that the cadherin/catenin system is present in choanoflagellates, suggesting that the cadherin/catenin system came into existence before the emergence of metazoans [34, 35]. Moreover, a polarized epithelium organized by beta- and alpha-catenins in *Dictyostelium discoideum*, which lacks a cadherin homolog, suggests that the function of catenins in cell polarity predates both classical cadherins and metazoan origins [36]. The highly conserved Hippo signaling pathway, which relays signals from adhesion receptors into the nucleus to regulate EMT and the expression of genes involved in cell proliferation, survival, and differentiation, also emerged before the origin of Metazoa [37]. Moreover, a nearly complete set of post-synaptic protein homologues with highly conserved protein interaction motifs has been discovered in sponges, another primitive colonial organism, which lacks neurons yet behaves and responds as one whole [38]. Altogether, these findings apparently indicate that neuronal synapses are evolutionary descendants of classical cell-cell adhesions and that the neuronal system may not be a necessary pre-requisite for coherent and coordinated behavior of a multicellular whole; coupled cytoskeletons may suffice.

Recapitulating phylogeny, neurons and epithelial cells descend ontogenetically from the same ancestor, the ectoderm. Therefore, it is fair to assume that the molecular mechanisms underlying neuronal plasticity and epithelial plasticity are similar and that the remodeling of neuronal networks, similar to the remodeling of epithelial architectures, relies on transient, localized, reversible, EMT-like transitions in neurons/neurites/synapses, which may or may not be accompanied and stabilized by changes in metabolism and gene expression. Irreversibly locking in any one of the metabolic/signaling/structural modes available to neurons/neurites would impair neurite/synapse remodeling and plasticity.

Furthermore, because NF-kB signaling and EMT-like transitions typically lead to “activated”, pro-migratory, pro-secretion phenotypes, which make cells to actively generate and secrete various chemokines, cytokines, and antimicrobial peptides, amyloid-beta, recently shown to be an antimicrobial peptide [39], may be naturally secreted by those disconnected neurons/neurites/synapses that have converted to a more “fluid”, pro-secretion phenotype, such as NF-kB-driven and/or amoeboid phenotypes. In fact, the cerebrospinal fluid from Alzheimer's disease patients was reported to contain antibodies that specifically recognize the amoeboid microglial cells in the developing rat central nervous system [40]. As AD progresses, an increasing number of remodeling neurons/neurites/synapses and/or microglial cells may become locked in pro-secretion, pro-migratory, pro-inflammatory modes/phenotypes, failing to reverse EMT-like transitions and to reconnect to the collective structure of neuronal networks, thereby undermining both cell- and tissue-level structures, functions, and performance. Such dynamics would naturally account for the increased secretion and accumulation of amyloid-beta, as well as for the increased levels of cytokines, chemokines, and their cognate receptors, chronic inflammation, oxidative stress, and neuronal degeneration observed in AD brains. In other words, similar to cancer, Alzheimer's disease can be described as a “never-healing wound” associated with chronically disconnected neurons/neurites/synapses.

Because contact inhibition is relieved in chronically disconnected neurons, they are expected either to try to proliferate, which would most likely lead to cell cycle arrest and apoptosis, due to their postmitotic nature, or to succumb to slow degeneration, depending on the particular signaling/metabolic mode/state in which they operate and/or the state of their microenvironment.

### On the role of neuronal microenvironment in AD pathogenesis

Nishimoto and colleagues proposed that TGF-beta promotes neuronal cell death by binding to APP and activating APP-associated signaling, and suggested that this could be one of the mechanisms of neuronal loss in Alzheimer's disease, as both neuronal and glial expression of TGF-beta are upregulated in AD brains [41, 42].

EpH4 cells are polarized, non-tumorigenic epithelial cells that form organotypic structures in 3D-collagen-gel cultures. When these cells are treated with TGF-beta, they undergo cell cycle arrest and apoptosis. However, when these cells stably express an oncogenic Ha-Ras, they undergo EMT in response to TFG-beta treatment and convert to a mesenchymal-like phenotype, which is then stabilized via an autocrine TGF-beta loop. When injected in mice, transformed EpH4-Ha-Ras cells rapidly undergo EMT in response to endogenous TGF-beta and form tumors. An expression profiling approach aimed at identifying molecular players involved in EMT and metastasis revealed that 13 out of the 75 genes that are upregulated in these cells upon EMT are NF-kB target genes [19, 43]. Moreover, NF-kB inhibition reverses EMT and abolishes the metastatic potential of EpH4-Ha-Ras cells *in vivo*. Constitutive activation of NF-kB in EpH4-Ha-Ras cells induces a partial EMT-like phenotype even in the absence of TGF-beta [19, 44].

Because Ha-Ras is known to promote aerobic glycolysis and to support adhesion-independent growth, it is fair to conclude that aerobic glycolysis and adhesion-independent growth may be necessary and sufficient to relieve the dependence of epithelial cells on their immediate environments significantly enough to allow for uncontrolled, adhesion-independent, TGF-beta-supported growth and migration.

It appears that Nishimoto and colleagues described essentially the same or similar mechanism of neuronal cell death that involves a TGF-beta autocrine loop, where A-beta acts as a factor that induces and promotes both neuronal and glial expression of TGF-beta [41]. It is fair to suggest that the TGF-beta expression induced by A-beta in neurons and glia in a given locality may indeed be detrimental to those neurons and/or glial cells that operate in a certain metabolic/signaling/structural mode. However, those neurons and/or glial cells that operate in the pro-secretory, pro-inflammatory, “NF-kB mode” and those cells that join them by undergoing an EMT-like transition in response to TGF-beta will be in fact supported and promoted by TGF-beta, similar to the case with transformed epithelial cells. One of the few options that are left to “TGF-beta-resistant” neurons/neurites to survive in such a microenvironment is to down-regulate neuronal TGF-beta receptor expression, which is indeed an early event in AD pathogenesis [45]. At the same time, reactive glia and activated macrophages, which operate in the “NF-kB mode”, are expected to upregulate both TGF-beta secretion and TGF-beta receptor expression (in addition to other growth factors, cytokines, and their receptors) and to move toward sources of A-beta and oxidative stress (“wounds”), thereby leading to the plaque-associated gliosis, inflammation, and the overall increased levels of TGF-beta in AD brains, which is also observed [46, 47], adding to confusion.

Given a large number of reports describing other APP ligands, growth factors, and cytokines that induce neuronal toxicity and neuronal and astroglial responses in a similar manner, it becomes difficult to avoid confusion. However, if we assume that variable and potentially innumerable triggers, causes, and means lead in fact to the same outcome, namely to a switch between alternative states/configurations of a cellular signaling/structural/metabolic network, then a great deal of confusion disappears. Indeed, similar to TGF-beta, NGF, BDNF, and other growth factors that promote APP-mediated neurite outgrowth and migration in such context as development and injury, act by activating Ras or its homologues, presumably to enable a switch to a proliferation-and-migration mode in neuronal/neurite functioning [48, 49]. In turn, Ras activation promotes APP expression and processing, which are associated with and/or required for the growth-factor-driven APP-mediated neurite outgrowth and migration [50, 51].

**Figure 5:**
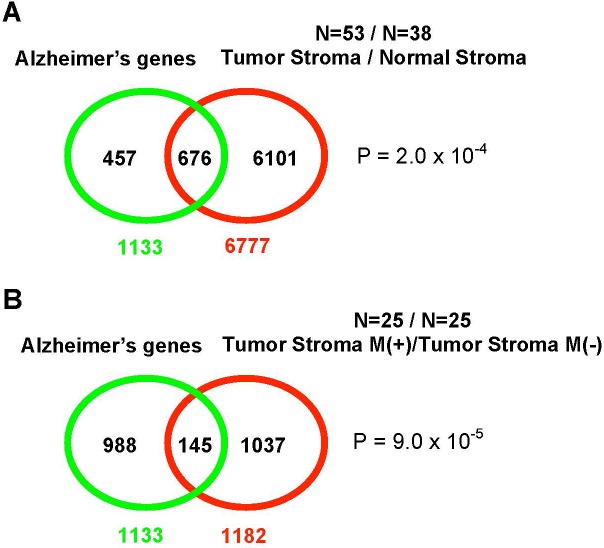
The overlap between the transcriptional profile of AD brains and gene expression in tumor stroma **A.** A Venn diagram shows the overlap of the AD brain transcriptional profile (53 patients) with the gene expression profile of tumor stroma obtained from breast cancer patients (38 patients); **B.** When the analysis is limited to the tumor stroma from metastatic cancer only (25 patients), the AD and tumor stroma transcriptional profiles show a slightly higher statistical significance in their overlap. “M” stands for metastasis. Reproduced and adapted from Pavlides S. et al. [52].

In consistency with the above inferences and conclusions, recent studies in the cancer field revealed an unexpectedly critical role of tumor microenvironment in promoting and supporting pro-migratory, pro-secretion, pro-inflammatory phenotypes in cancer cells [52, 53]. A system-scale analysis of gene expression in the tumor-associated stroma isolated from human breast cancer patients revealed that tumor stroma is characterized by constitutively active TGF-beta signaling and the ability to promote EMT in normal mammary epithelial cells. The major sets of genes that are upregulated in the tumor-associated stromal fibroblasts, as compared to their normal counterparts, are glycolysis genes (19), mitochondrial genes (233), oxidative stress genes (51), HIF-1 target genes (213), and NF-kB target genes (199). Surprise came when it was discovered that this transcriptional profile spectacularly overlaps with the transcriptional profile of AD brains [52] (Figure 5).

## CONCLUSIONS

As constituents of a continuously remodeling tissue, neurons may undergo localized, transient, reversible, EMT-like, organizational transitions in response to a variety of physiological and non-physiological stimuli, such as growth factors, hormones, neuronal/brain stimulation/activity, learning, diet, physical activity, sleep, drugs, alcohol, smoking, hypoxia, and inflammation. In all likelihood, reversible organizational transitions occur continuously on multiple scales of space and time in synapses, neurites, neurons, and neuronal networks to enable neuronal plasticity, learning, memory, and cognition. They are expected to occur continuously throughout life throughout the body, with an especially high frequency and/or extent during developmental stages, active learning, regeneration, sleep-wake cycles, and in the regions of high neuronal plasticity/restructuring. However, when the (APP-associated?) signaling/metabolic network that controls these transitions is dysregulated, neurons/neurites/synapses may become locked in cancer-like, pro-secretion, pro-inflammatory modes/phenotypes, which are unable to support stable tissue architecture, thus leading to a progressive impairment of synaptic and neuronal functions, the accumulation and deposition of A-beta and neurofibrillary tangles, and impaired learning, progressive loss of memory, and cognitive decline. Similar to tumorigenesis, AD pathogenesis is expected to progress slowly and imperceptibly over time, accelerating non-linearly in the elderly and/or during the last stages of the disease, as an avalanche.

## DISCUSSION

The presented analysis suggests the existence of evolutionarily conserved molecular mechanisms that enable the emergence of stable and coherent yet reconfigurable and adaptable multicellular architectures/networks, such as tissues, neuronal networks, and multicellular colonies. The emergence, persistence, and stability of coherent multicellular architectures appear to involve the stabilization of cytoskeletal networks in individual cells, concomitant with the coupling of individual cytoskeletons via cell adhesions into an integrated, system-wide, cytoskeletal organization that defines the structure, stability, and performance of a multicellular whole. The breakdown, destabilization, or mal-performance of cytoskeletal structures, cell adhesions, or ECM is expected to undermine the structure, stability, and coherence of the multicellular whole. However, to develop and to adapt to stress and change, any multicellular system/network must retain a fair degree of organizational plasticity. The two essential properties that are required for the survival and prosperity of coherent multicellular networks/architectures in conditions of stress and unpredictable change – organizational stability and plasticity – are mutually contradictory. Multicellular systems appear to resolve the contradiction by undergoing reversible organizational transitions, which may potentially encompass a part of a cell, a cell, a group of cells, or a multicellular system/network. In such contexts as development, regeneration, wound healing, adaptation, and learning, the remodeling and reorganization of multicellular systems/networks appear to rely on reversible, EMT-like, organizational transitions, which may or may not be accompanied and/or stabilized by changes in metabolism and gene expression.

In the context of the CNS and neuronal networks, neurite outgrowth and synaptogenesis, accompanied by concurrent stabilization of neuronal cytoskeletons and adhesions/synapses, lead to the formation of properly wired, stable and coherent neuronal architectures. As postulated herewith, EMT-like organizational transitions represent a mechanism that allows for continuous neuronal plasticity and remodeling of neuronal networks throughout life, thus enabling and supporting learning, memory, and cognition. The hypothesized EMT-like organizational transitions may potentially encompass a part of a synapse, a synapse, a part of a neurite, a neurite, a neuron, or a group of interconnected neurons, mediating correspondingly synaptic plasticity, neurite plasticity, and large-scale plasticity of neuronal networks. The EMT-like transitions and structural reorganization involving neuronal cytoskeletons and adhesions/synapses can be induced both by physiological processes and signaling and by non-physiological stresses, thus coupling and integrating the internal and external processes to enable interactive learning and continuous co-evolution of the external and the internal. As long as EMT-like organizational transitions remain reversible and two counter-opposing processes – disconnection-and-retraction and proliferation-and-reconnection – are not impaired but dynamically balanced, a multicellular network can survive, adapt, and prosper in conditions of significant stress and continuous change. However, the chronic impairment and/or dysregulation of reversible organizational transitions in synapses, neurites, neurons, and/or neuronal networks, which lead to a persistent imbalance in the disconnection-and-retraction versus proliferation-and-reconnection dynamics, impair neuronal plasticity and connectivity, leading to neurodegeneration. Chronic impairment/dysregulation of reversible, EMT-like, organizational transitions can be potentially caused by many different factors, including but not limited to inherited or acquired genetic mutations, epigenetic modifications, metabolic disorders, endocrine disorders, hormonal imbalance, head trauma, deregulated cell signaling, inadequate diet, environmental pollutants, misguided behaviors and habits, and combinations thereof.

**Figure 6:**
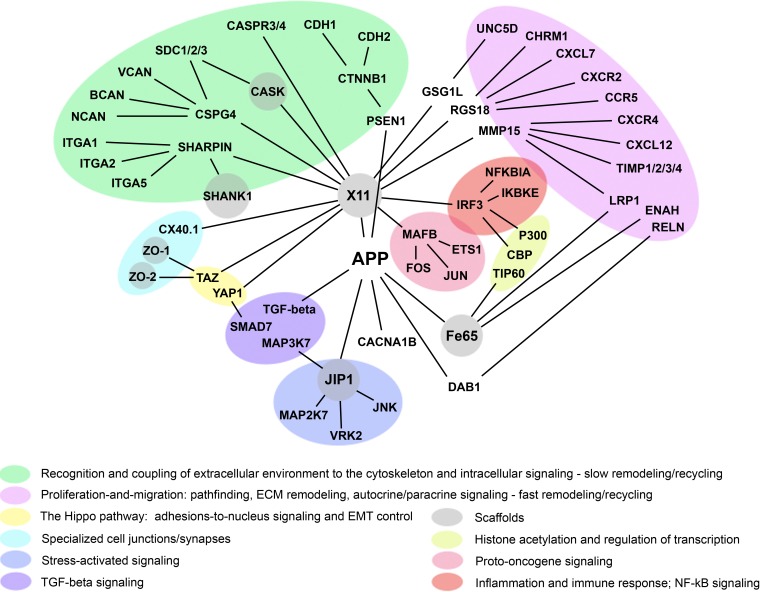
APP connectome, cell adhesions, and cell signaling The shown APP-associated network of protein interactions may potentially couple and coordinate the indicated cellular functions and cell signaling (color-coded). Brief descriptions of the shown proteins are given in Table 1 (Supplemental Data).

The model outlined above allows one to rationalize the branch of APP connectome that couples neuronal adhesions/synapses, cytoskeletal dynamics, and cell signaling (Figure 6 and Table 1 in Supplemental Data), assuming that APP-associated complexes are dynamic, steady-state, multiprotein entities whose composition is not fixed but changes in space and time in a combinatorial-like fashion, with a relatively stable “core” of constituents and a varied “periphery”. According to this view, the composition of APP-associated complexes is not constant but depends on the context, e.g., immature versus mature neurons, growth cones versus synapses, the plasma membrane versus endosomes or ER, or the cell nucleus. Moreover, within a given context, the composition of APP-associated complexes also changes in response to changes in extracellular and intracellular conditions and signaling. This assumption is a straightforward generalization of the dynamic nature of sub-cellular organization revealed by advanced, fluorescence-based, imaging technologies, which allowed for the observation and quantitative analysis of molecular dynamics in living cells. Virtually all of the supra-molecular structures and complexes examined thus far, which include but not limited to nucleoli, splicing factor compartments, nuclear pore complexes, euchromatin, heterochromatin, the cytoskeleton, the Golgi complex, and the multi-protein complexes that mediate basic biological processes, such as DNA replication and repair, transcription, signaling, and metabolism, have been found to exist in living cells as steady-state, continuously remodeling, multi-protein architectures, which dynamically respond to changes in extracellular and intracellular conditions and signaling [54, 55].

Given the inherently dynamic, adaptive, combinatorial-like nature of multi-protein complexes in living cells and the significant functional substitutability of their constituents, the same or functionally similar processes in the cell can be potentially realized by different and multiple means. Therefore, in addition to the situation- and context-dependent means by which this or that cellular process/function is actualized, it may be also useful to analyze the conserved interrelations among conserved cellular processes/functions that allow biological systems/networks to harmoniously combine stability and exquisite specificity, on the one hand, and organizational plasticity, on the other. In this regard, APP connectome suggests that the proteins closely associated with APP can be conditionally divided into three main classes. One class comprises proteins that in most, but not all, studied contexts promote cell proliferation and migration, often by disrupting or weakening cell adhesions and multicellular integration (e.g., TGF-beta, growth factors, cytokines, and proto-oncogenes), whereas the second class comprises proteins that exert opposite effects, i.e., inhibit cell proliferation and migration, often by promoting cell adhesion and integration (e.g., cadherins, integrins, cell junction scaffolds, and tumor suppressors). The third class comprises proteins that, depending on the context and/or isoform, elicit both, mutually opposing, effects (e.g., APP, ADAMs, and SMADs).

It is worth noting that, although, some of the proteins that are closely associated with APP may seem at first glance to have little or no relevance to APP possible functions and/or AD pathogenesis, more often than not, analysis of research literature proves the first impression to be misleading. As an example, at first glance, the CXCR4, CXCR2, and CCR5 chemokine receptors and their cognate ligands, SDF-1/CXCL12 and CXCL7, which are well-known and essential mediators of chemotaxis in cells of the immune system, may appear as rather unexpected members of APP functional connectome. However, an analysis of recent research literature reveals a different perspective. The CXCR4 receptor and its cognate ligand SDF-1/CXCL12 are believed to be the most ancient, “original”, chemokine-receptor pair that emerged in evolution before the emergence of the elaborated immune system, with the main function in developmental morphogenesis. Widely expressed in the developing embryo, the CXCR4 receptor and its ligand SDF-1/CXCL12 are essential for developmental morphogenesis of many tissues. In the developing nervous system, SDF-1/CXCR4 signaling controls axon morphogenesis and directs neuronal migration and axonal pathfinding. In the adult brain, SDF-1/CXCL12 is thought to influence neurogenesis as well as recruitment of brain resident and non-resident circulating cells toward sites of lesion [56, 57]. The CCR5 and CXCR4 are two primary co-receptors for HIV-1 entry and potential mediators of neuropathogenic effects caused by the HIV virus and/or its products, and, possibly, by other viruses, such as Cytomegalovirus (CMV) and the Epstein-Bar virus (EBV) [57]. Mechanistically, chemokine receptors such as CXCR4 appear to link extracellular stimuli to motor proteins, cytoskeletal networks, and mitogenic signaling inside cells [58, 59]. Pertinently, the CXCR2 and CXCR4 chemokine receptors are currently among the most promising GPCR targets in anti-cancer drug development, as they are aberrantly expressed in many tumors, where they exert potent proliferative, pro-survival, and pro-migratory effects [60, 61]. Remarkably, the reverse may also be true, and familiar neuronal proteins, with seemingly established functions, can play unexpected roles in different contexts. As an example, corneal epithelial cells (CEC) in wounded cornea and small cell lung carcinoma cells express both nicotinic and muscarinic acetylcholine (ACh) receptors and use acetylcholine and as an autocrine and paracrine chemokine and growth factor. In the case of CEC, constant stimulation of CEC via both muscarinic and nicotinic signaling pathways was essential for CEC survival and directional and random migration in vitro. Both alpha-7 and non-alpha-7 nAChRs elicited chemotaxis, with the alpha-7 signaling exhibiting a stronger chemotactic effect. Cholinergic stimulation of CEC upregulated the expression of the integrins and cadherins involved in epithelialization [62]. Muscarinic ACh receptors, subtypes M1, M3, and M5, were defined as conditional oncogenes, due to their high transformation potential [63], whereas mouse mutants for the nicotinic acetylcholine receptor beta-2 subunit display altered expression of genes involved in cell adhesion, calcium signaling, and neurodegeneration [64].

The concept of reversible, EMT-like, organizational transitions, together with the concept of a dynamic balance between disconnection-and-retraction and outgrowth-and-reconnection, appear to provide a unifying conceptual and mechanistic framework that has a potential to harmoniously integrate many of the cellular processes known to be involved in AD pathogenesis. For example, the regulated and limited proteolysis of adhesion receptors, adapters, and/or associated cytoskeletal structures by secretases, sheddases, calpains, and/or caspases in response to transient calcium influx and/or kinase/phosphatase activation, which are commonly associated with activation of cell surface receptors for growth factors, chemokines, ECM components, glucose, amino acids, and lipoproteins, may constitute a general mechanism that enables chemotaxis, pathfinding and directed migration/outgrowth of cells, neurites, and axons via localized, reversible, structural transitions. Shifting a dynamic balance toward outgrowth-and-reconnection, via progressive cytoskeletal stabilization/”freezing” in transiently “melted”, i.e., activated, regions at the cell surface, would lead to a migration/outgrowth toward “attractive” ligands and environments, along corresponding gradients and/or paths, via localized “melting-freezing” cycles. Shifting a balance in the opposite direction, toward disconnection-and-retraction, via cytoskeletal and/or adhesional destabilization, would lead to repulsion and/or change in the direction of migration/outgrowth. A dynamic balance between exocytosis and endocytosis of cell surface receptors, accompanied by receptor sorting and recycling, especially when targeted to the same activated sites at the cell surface, can be interpreted as an integral part of the same process. The intrinsic dynamic instability of microtubules, manifested as repetitive cycles of microtubule polymerization and extension and avalanche-like depolymerization and contraction, whose frequency and extent are regulated via posttranslational modifications, such as phosphorylation and acetylation, and/or reversible interactions with molecular motors and/or microtubule-binding proteins, may also be an integral part of the same plasticity mechanism. Limited and regulated apoptosis, a form of programmed cell death responsible for developmental and regenerative morphogenesis, and autophagy may function as mechanisms that mediate cellular and tissue restructuring/plasticity on larger scales. Other intracellular proteolytic systems, such as ubiquitin/proteasome system and lysosomes, may also be integral parts of the reversible, EMT-like, organizational transitions that underlie neuronal plasticity.

Already in its present form, the evolving AD model appears to assimilate and integrate a large body of diverse observations pertaining to AD pathogenesis in a self-consistent and mutually reinforcing manner. Indeed, APP and presenilin 1, two proteins that are mutated in the early-onset familial Alzheimer's disease and that were used to generate common AD animal models, play central roles in the CNS morphogenesis, neuronal migration, neurite outgrowth, synaptogenesis, and synapse maintenance and remodeling [65-68]. The formation and stabilization of neuronal adhesions/synapses and the regulated assembly/disassembly and turnover of adhesions/synapses in response to the activation of various ion channels and receptors for neurotransmitters, growth factors, guiding molecules, cytokines, nutrients, and xenobiotics appear to be essential cellular functions of these proteins [25, 69-73]. Indeed, approximately ten years ago, expressing the evolving consensus in the field, a leading AD researcher defined Alzheimer's disease as a synaptic failure [74].

However, in addition to the established importance of neuronal adhesions/synapses in the AD pathogenesis, the evolving AD model places an equal, if not higher, importance on structural transitions in neuronal cytoskeletal networks, which may or may not be accompanied and/or stabilized by changes in metabolism and gene expression. Moreover, the model implies that adhesion/synapse activity and cytoskeletal dynamics (as well as metabolism and cell signaling) are intimately interdependent. Therefore, the proteins that couple cytoskeletal networks to the adhesion/synaptic machinery, such as the highly conserved cadherin/catenin system, and the proteins that couple neurons to their extracellular milieu, such as cadherins, syndecans, neurexins, integrins, and various other receptors for extracellular stimuli, play a major regulatory role in the plasticity, survival, and performance of synapses, neurites, neurons, and neuronal networks/architectures. Indeed, E-cadherin knockout mice, N-cadherin knockout mice, and Presenilin 1/2 double-knockout mice die during early embryonic stages, displaying major disorganization of cellular architectures [75-79]. Conditional knockout of N-cadherin in the developing cortex leads to randomization of the ordered arrangement of cells in the intra-cortical structures, presumably due to the impairment of adherens junctions in developing neuroepithelial cells, whereas in the mature brain N-cadherin is believed to play an important role in LTP, learning and memory by linking synaptic activity and synaptic plasticity [76]. Because the organization and dynamics of cytoskeletal networks is a relatively understudied area of research, which is thus less commonly known and/or appreciated by AD and cancer researchers, it may be useful to briefly summarize recent research highlights in this area.

The cytoplasm of animal cells is structured and supported by a scaffolding composed of actin microfilaments, intermediate filaments, and microtubules, three major fibrous biopolymers of the cell that form three distinct yet interdependent filamentous systems. All three exists as dynamic, continuously remodeling, frameworks/networks, which are involved in virtually every aspect of cellular physiology.

**Figure 7:**
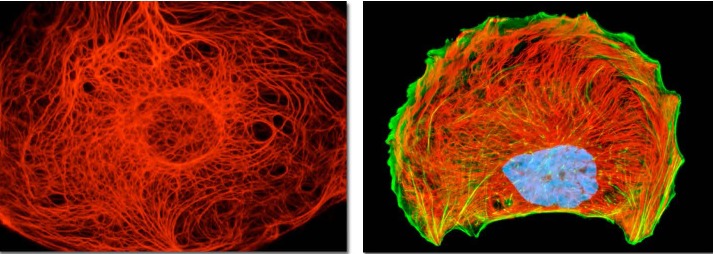
Cytokeratin and vimentin intermediate filament networks in cultured cells Cell images illustrate the organization of keratin (the left image) and vimentin (the right image) cytoskeletal networks in cultured cells. Together with actin microfilaments and microtubules, intermediate filaments mediate dynamic scaffolding and organization of intracellular space and cellular functions. Intermediate filament networks emerged recently as versatile, dynamic platforms that enable the cell-wide integration and coordination of cell structure, signaling, and metabolism (see text for details). Images courtesy of Olympus America.

Intermediate filaments (IFs), such as cytokeratins, vimentin, neurofilaments, glial fibrillary acidic protein (GFAP), and nuclear lamins, form dynamic, sponge-like, cytoskeletal networks that support sub-cellular organization and couple various intracellular structures and processes in living cells, including actin- and tubulin-based cytoskeletons, mitochondrial network, plasma membrane, and internal membrane systems, such as endosomes, lysosomes, ER, and Golgi, as well as the organization and dynamics of chromatin and transcription in the nucleus (Figures 3 and 7) [80-82]. Because IF networks integrate actin- and tubulin-based cytoskeletons, cellular membranes, organelles, and chromatin into large supra-molecular assemblies, which behave as coherent wholes, dynamic IF networks are well suited to perform as integrative devices/frameworks that coordinate structural and functional plasticity on a variety of scales, from sub-cellular scales to tissue-level organization. Consequently, controlling the organization and dynamics of IF networks provides a convenient and sensitive means to regulate and coordinate supramolecular-, cell- and tissue-level structure, function, and performance [81].

Unlike the highly conserved constituents of actin filaments and microtubules, IF proteins are encoded by one of the largest gene families in humans, comprising more than 67 different genes, whose products are assembled in a combinatorial-like fashion to form different IF networks. The expression of IF genes is context-dependent and cell- and tissue-specific, bearing an obvious relationship to cell differentiation and providing each major cell type with a relatively specific IF “fingerprint” [80-82]. Intermediate filaments are almost exclusively associated with multicellularity and cell differentiation and any drastic impairment of IF function appears to be incompatible with cell viability. There are over 30 human disorders caused by genetic mutations in intermediate filaments. IF mutations are typically associated with degeneration, dystrophies, progerias, and extreme fragility upon stress, i.e., with the properties typically associated with aging. Examples of neuronal diseases caused by IF mutations include familial and sporadic amyotrophic lateral sclerosis (ALS), infantile spinal muscular atrophy, and hereditary sensory motor neuropathy [83, 84].

Neurons and epidermal keratinocytes are two cell types in which IFs are most abundant (up to 85% of total protein) [85]. Neurofilaments (NFs), a major type of intermediate filaments in neurons, are a major determinant of cyto-architecture in neurons, defining axonal, dendritic, and overall neuronal morphologies [86]. Axonal microtubules are imbedded into a structured network of neurofilaments that couple microtubules, organelles, vesicles, auxiliary proteins, and lipid membranes into one functional whole. Intermediate filaments are major phosphoproteins of cells. Reversible structural transitions in IF networks are sensitive to a dynamic balance between phosphorylation and dephosphorylation of IFs, which is controlled by specific kinases and phosphatases. Recent phosphoproteomics studies revealed a significant number of proline-directed Ser/Thr sites that are differentially hyperphosphorylated in neurofilaments from AD brains, suggesting the involvement of proline-directed kinases, such as Cdk5, glycogen synthase kinese 3 beta (GSK3beta), ERK1/2, p38 MAP kinase, or stress-activated protein kinases (SAPK/JNK), and PP2A phosphatase in the regulation of the organization and dynamics of NF networks [87-89]. Deregulation of these kinases, such as hyperactivation of Cdk5 in response to neuronal insults (e.g., oxidative stress, A-beta toxicity, glutamate toxicity), leads to accumulation of hyperphosphorylated cytoskeletal proteins in neuronal perikarya, tangles of phospho-tau, and aggregated phosphorylated neurofilaments, pathological hallmarks of AD, ALS, and dementia with Lewy bodies [90, 91]. The similar processes of reversible IF networks collapse, protein aggregation, and a recovery, following removal of insult, were observed in living epithelial cells treated with okadaic acid, a phosphatase inhibitor and a tumor promoter [92].

The intermediate filament vimentin is commonly used as an EMT marker, and EMT in epithelial cells is associated with down-regulation of cytokeratin expression and up-regulation of vimentin expression. Vimentin is also a marker of astrocyte activation. Vimentin expression is high and ubiquitous during early developmental stages, and down-regulated along with the progress in cell differentiation and organism development, giving way to cell-type-specific IF gene expression. In the developing CNS of the chick and rodents, vimentin is expressed by virtually all cells within the proliferative neuroepithelium, including neuronal precursors where its expression is necessary for neurite extension, as a component of cytoskeletal assembly [89, 93-95]. In adult organisms vimentin expression is abundant only in certain cells and cell types, notably in motile cells and cells that function in the conditions of continuous stress and/or change, such as fibroblasts and vascular endothelial cells. Vimentin expression is commonly up-regulated upon cell “activation” in such contexts as regeneration, wound repair and cancer, and in activated macrophages and glia. Mechanistically, vimentin expression is regulated by growth factors, such as TGF-beta, PDGF, EGF, and FGF, and cytokines, whose signaling is mediated by SMADs, c-Jun, c-Fos (AP-1), and Sp1/Sp3 transcription factors [96].

Vimentin and cytokeratin networks exhibit drastically different organization and dynamics in living cells. Both vimentin and cytokeratin form dynamic, motile, steady-state networks that continuously change their configurations. However, the rates of subunits exchange in vimentin fibrils and translocation of vimentin structures are more than an order of magnitude faster than the corresponding rates in keratin IF networks, suggesting that vimentin networks may support more “fluid” intracellular organization with faster dynamics [82, 97]. Although a proper functioning of both networks requires functional microtubules, the overall organization of keratin IF networks remains relatively unaltered after treatment of cells with microtubule inhibitors, whereas vimentin IF networks are dramatically reorganized upon microtubule disruption, collapsing primarily into a perinuclear cup and intracellular aggregates [82, 98]. Vimentin IF networks appear to rely on fast motors, such as kinesins and dyneins, whereas keratin networks may largely rely on slow motors, such as myosins. Correspondingly, epithelial cells, in which cytokeratin is the major IF protein, move at rates that are more than an order of magnitude slower than that of fibroblasts, in which vimentin is the major IF protein [82]. Also, certain cytokeratin structures undergo continuous flow-like movement largely from the cell periphery toward the cell nucleus, whereas the corresponding vimentin structures move mostly in the opposite direction, i.e., from the perinuclear region toward the plasma membrane [86, 98].

A recent analysis of the neuronal expression of vimentin in AD brains revealed that vimentin was localized to neuronal perikarya and dendrites in AD affected areas of the brain, with vimentin-immunopositive neurons prevalent in regions exhibiting intra- and extracellular A-beta deposition. Neuronal co-localization of vimentin and A-beta was common in the cerebral cortex, cerebellum and hippocampus. In addition, it was found that neurons in human fetal brain express vimentin concurrently with periods of rapid neurite extension. Vimentin expression was also detected in neurons in affected brain regions of AD transgenic (Tg2576) mice and in brain tissue subjected to mechanical injury. Consequently, the authors of the study suggest that “…neurons express vimentin as part of an evolutionarily conserved, damage-response mechanism which recapitulates a developmental program used by differentiating neurons to establish dendrites and synaptic connections” [99]. A recent phosphoproteomics study of neurofibrillary tangles (NFTs) revealed neurofilaments and vimentin as integral constituents of NFTs, suggesting their potential involvement in the NFT genesis [88]. Neurofilaments are the main constituent of Lewy bodies, protein aggregates found in a number of neurodegenerative diseases, including Parkinson's disease and Lewy Body dementia. Aberrant neurofilament accumulation are also the pathological hallmark of other neuropathies, such as amyotrophic lateral sclerosis (ALS), Charcot-Marie tooth disease type 2 (CMT2), progressive supranuclear palsy, and giant axonal neuropathy [83, 100], suggesting that dysregulation of neurofilament networks may be a common feature shared by neurodegenerative disorders.

Altogether, a large and diverse body of experimental evidence suggests a key role of IF cytoskeletal networks and their organizational dynamics, including EMT-like transitions, in the pathogenesis of AD and other degenerative disorders. It is worth noting that the intimate interdependence between the activity/dynamics of adhesions/synapses and the organizational dynamics of cytoskeletal networks provides a potential mechanistic link between the APP/A-beta activity in neuronal adhesions/synapses and the genesis of neurofibrillary tangles in neuronal processes and bodies.

To evaluate the “practical worth” of the evolving AD model and to demonstrate that, more often than not, the experimental evidence needed to validate and/or refine an evolving model is already present in research literature and databases, let us conduct an experiment *in charta* and briefly investigate if there is any support for one of the key findings/predictions generated in the course of the AD model evolution, namely a putative switch of cells in AD brains to cancer-like, pro-secretion, pro-inflammatory phenotypes.

The emergence and persistence of a cellular phenotype implies the existence of environmental pressures leading to its acquisition as well as the existence of molecular mechanisms that confer stability to the phenotype. Two well-known molecular mechanisms that enable stability of acquired cellular phenotypes are somatic mutations and epigenetic modifications, such as DNA methylation and histone acetylation.

A brief search for the research literature related to somatic mutations in Alzheimer's disease reveals that 65% of examined Alzheimer's brains harbored the T414G mutation in the mtDNA control region (CR), whereas this mutation was absent from all controls. Moreover, all AD brains had an average 63% increase in heteroplasmic mtDNA CR mutations and AD brains from patients 80 years and older had a 130% increase in heteroplasmic CR mutations. AD brains also exhibited an average 50% reduction in the mtDNA/genomic DNA ratio and in the mitochondrial ND6 (Complex I) transcripts, as compared to controls, suggesting an acquired and persistent impairment of oxidative phosphorylation in sporadic AD [101]. As mentioned earlier, chronic impairment of oxidative phosphorylation is expected to lead to a chronic shift to more “fluid”, de-differentiated cellular phenotypes, as was first proposed to be the case for cancer cells by Warburg [16]. Beck et al. reports a case of sporadic early-onset AD attributed to a somatic mosaic presenilin-1 mutation in the brain [102]. However, although the association of somatic mutations with a variety of neurological disorders enjoys a substantial and growing experimental support, the field as a whole appears to be at its beginnings, in part because such mutations are difficult to detect, as they tend to be brain- or even cell-group-specific and mosaic, often arising due to the inherited heterozygous mutations that acquire a somatic mutation (a “second hit”) only in selected cells or cell populations [103]. Nevertheless, the existence of environmental pressures leading to the acquisition and persistence of somatic mutations that result in neuropathology appears to be a sound and experimentally validated concept.

A brief search for and analysis of the research literature pertinent to epigenetic modifications in Alzheimer's disease reveals a large body of evidence that unambiguously implicates epigenetic modifications in AD pathogenesis, in fact too large to even summarize it here. However, a few relevant observations are worth mentioning.

A large body of experimental evidence indicates that chromatin modifications, in particular histone acetylation, are critically involved in learning, memory, and plasticity. Increased histone acetylation accompanies memory formation, whereas decreased histone acetylation (transcription-repressive context) accompanies memory impairment. Inhibitors of histone deacetylases (HDACs) facilitate learning and memory in wild-type mice and in mouse models of neurodegeneration [104-109]. Elevated levels of HDAC2 deacetylase may be a common feature of neurodegenerative diseases, including Alzheimer's disease [110, 111]. Consequently, HDAC inhibitors are actively discussed as promising therapeutics for chronic CNS disorders and acute injuries [112-114]. In regard to Alzheimer's disease, HDAC inhibitors exhibit various beneficial effects in mouse models of AD, such as APP/PS1 mouse models of familial AD [115, 116], and the CK-p25 mouse model of AD-related neurodegeneration and memory decline [109].

Perhaps not coincidentally, as the presented AD model suggests, HDAC2, whose elevated levels are commonly associated with neurodegenerative disorders, is an oncogene, whereas HDAC inhibitors are considered as a new promising class of anti-cancer drugs [114, 117]. Currently, there are more than a dozen of HDAC inhibitors in various stages of clinical development, mostly in phase I/II clinical trials for a variety of cancers, including valproate, which is in phase III trials for cervical cancer and ovarian cancer [118]. Valproate is an anticonvulsant and mood-stabilizing drug, commonly used to treat epilepsy, bipolar disorder and migraine headaches. Valproate was discovered to act as a HDAC inhibitor [119]. More recently, valproate was discovered to reopen plasticity in the adult brain, allowing for acquisition of perfect pitch by adults, the ability that is normally restricted only to a certain critical period early in life [120]. In fact, a number of the same compound classes and even the same compounds are being evaluated as promising drug candidates both in anti-cancer and anti-AD drug development. In addition to HDAC inhibitors such as sodium butyrate and valproate, examples include docosahexaenoic acid (DHA), SirT1 activators, such as resveratrol and curcumin, and NF-kB inhibitors, such as ashwaganda and withaferin A. It is worth noting that the rationale behind using NF-kB inhibitors in cancer therapy comes from the multiple studies demonstrating that inhibiting NF-kB signaling in various model systems, either by genetic or pharmacological means, blocks or reverses EMT induced by NF-kB or other factors, suppresses pro-inflammatory phenotypes, and inhibits proliferation, migration, and invasion of cancer cells [19, 121, 122]. It is also worth noting that withaferin A targets the type III intermediate filaments vimentin, GFAP, and desmin, potently disrupts IF networks, downregulates injury-induced vimentin expression, and blocks TGF-beta-induced transformation via cell cycle arrest [123, 124]. Correspondingly, withaferin A is considered as a promising anti-cancer, anti-fibrosis, and anti-gliosis pharmacological agent [123, 125, 126].

Although DNA methylation has been long appreciated as a major epigenetic mechanism used by cells to modify and stabilize their gene expression profiles, recent advances in high-throughput technologies and methods enabled a quantum leap in the scope and precision of the DNA methylation analysis. Genome-wide analyses of CpG dinucleotides (DNA methylation sites) have revealed that the DNA methylome evolves in the course of life and that DNA methylation levels at certain CpG sites can be highly variable across individuals and stable over time in an individual [127-129]. These observations suggest that individual-specific DNA methylation patterns at certain loci may reflect individual experiences and traits, including predispositions to and/or risk factors for specific diseases. Therefore, the identification and analysis of differentially methylated regions (DMRs) in diseased versus healthy individuals may help to uncover disease-promoting genes and/or disease-associated DNA methylation patterns. One of the most striking alterations discovered in the aging DNA methylome is the emergence of regions of age-associated hypo- and hyper-methylation, which bear a significant resemblance to the DNA methylation patterns found in age-associated diseases such as cancer [127, 130]. The age-related changes in DNA methylation are now thought to be a major contributor to carcinogenesis [131-133].

**Figure 8:**
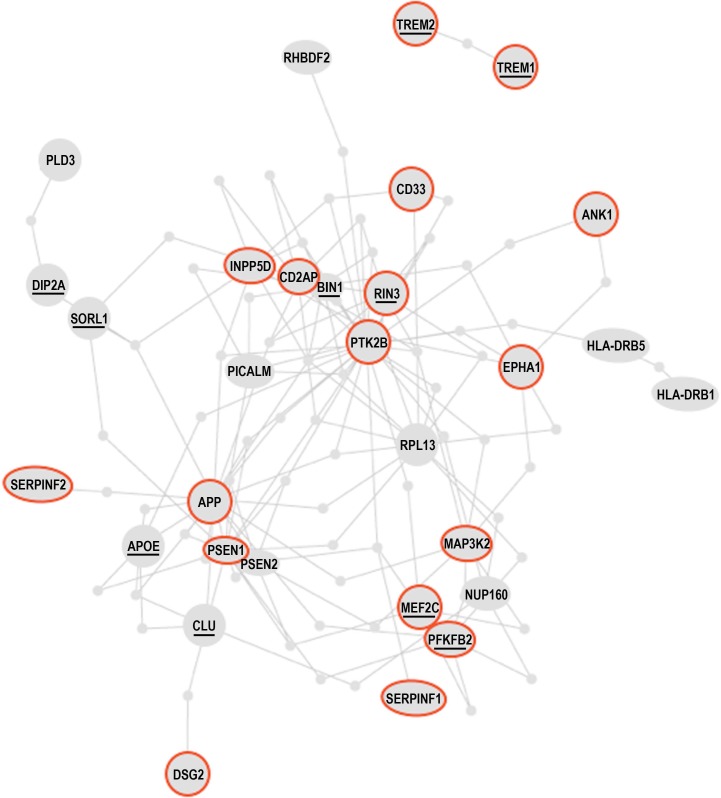
The Alzheimer's disease susceptibility network Products of the AD susceptibility genes and products of the genes that are differentially methylated in AD patients form a “tightly-knit” protein interaction cluster, being interconnected via at most one shared interacting partner. The resulting AD susceptibility network is discussed in the text. Brief descriptions of the proteins circled red are given in Table 2. Underlined proteins are discussed in the text. Adapted from [129] by permission from Macmillan Publishers Ltd: Nature Neuroscience; De Jager, PL et al., Nat Neurosci 17(9):1156-63; ©2014.

A recent genome-wide study found that the methylation level at 71 of the 415, 854 interrogated CpG sites significantly correlated with the burden of neuritic amyloid plaques in the 708 assessed brain autopsies. Eleven of the 71 differentially methylated CpGs were confirmed using Braak staging, a different quantitative measure of AD pathology. The analysis of expression levels of the genes found in the vicinity of the validated differentially methylated regions in AD brains led to the discovery of 7 genes, whose expression is altered in AD, presumably due to differential methylation. These are *ANK1, CDH23, DIP2A, RHBDF2, RPL13, SERPINF1 and SERPINF2* [129]. The authors of the study went on further and constructed the AD susceptibility network, by combining proteins encoded by the known AD susceptibility genes, derived from genome-wide association studies of genetic polymorphism associated with AD, and proteins encoded by the newly discovered genes that are differentially methylated in AD. Remarkably, the products of the known AD susceptibility genes and the products of the genes that are differentially methylated in AD form a “tightly-knit” protein interaction cluster, being interconnected via at most one shared interactor [129] (Figure 8). This fact suggests that AD pathogenesis may involve few key physiological processes that are closely interrelated. Indeed, the genes whose products are involved in the same or closely interdependent complexes and/or processes are more likely to co-evolve both on phylogenetic timescales, e.g., via acquisition of genetic alterations/mutations, and on ontogenetic timescales, e.g., via acquisition of epigenetic modifications, such as DNA methylation.

In Figure 8 and the accompanying Table 2 we reproduce the published AD susceptibility network and provide brief annotations describing some of the proteins comprising the network. Approximately 50% of proteins comprising the AD susceptibility network are involved in cell adhesions, EMT, cytoskeletal coupling, and the associated signaling that controls cell proliferation and migration. Two proteins, TREM1 and TREM2, are involved in acute and chronic inflammation, correspondingly. Because TREM1 and TREM2 are the only two proteins that are separated from the main cluster, they may relate to non-neuronal cells involved in the immune response and inflammation associated with AD. MEF2 and PFKFBP proteins regulate oxidative phosphorylation and glycolysis, correspondingly. The presence of these proteins in the AD susceptibility network supports the hypothesis of a metabolic shift from oxidative phosphorylation to glycolytic modes of energy production in AD brains. Remarkably, both the somatic mtDNA mutations detected in AD brains (described above) and the AD-associated genetic polymorphism of the MEF2 loci, appear to target the same protein, the same complex, the same process, and the same organelle, namely the ND6 subunit, NADH dehydrogenase (Complex I), oxidative phosphorylation, and mitochondria, correspondingly [101, 129, 138], (Table 2). Moreover, whereas MEF2 controls NADH dehydrogenase, which is the “entry enzyme” of oxidative phosphorylation, PFKFBP (6-phosphofructo-2-kinase/fructose-2, 6-biphosphatase) controls the “entry” of the glycolytic pathway. PFKFBP (isoforms PFKFBP1-4) is a conserved bifunctional enzyme that sets the rate of glycolysis in cells by controlling intracellular levels of fructose-2, 6-biphosphate (F2, 6BP). F2, 6BP is the most potent allosteric activator of PFK-1 (6-phosphofructo-1-kinase), which catalyzes the essentially irreversible, commitment step in the glycolytic pathway. PFK-1 is inhibited by ATP and activated by ADP. However, F2, 6BP allows PFK-1 to bypass the inhibition by ATP, thereby permitting cells to maintain high rates of glycolysis even at physiological ATP concentrations. In part, this mechanism may potentially account for the Warburg effect, a high rate of glycolytic flux in the presence of oxygen. PFKFBPs are markedly induced in response to hypoxia and hypoxia mimics via hypoxia-sensitive transcription factors, such as the HIF-1 complex [139, 140]. PFKFBPs are also upregulated in response to growth factors and hormones, such as insulin and progesterone [137, 141]. PFKFBPs are overexpressed in lung, breast, colon, prostatic, pancreatic, and ovarian adenocarcinomas, and PFKFBP up-regulation parallels the up-regulation of glycolytic enzymes [137]. The transformation of cells by oncogenic Ras, which leads to aerobic glycolysis and permits adhesion-independent growth, requires PFKFBP activity [142]. RIN3, another member of the AD susceptibility network, is an effector of activated Ras, which may control and coordinate cell adhesion and migration by coupling and coordinating endocytosis of activated receptors and cytoskeletal remodeling [143]. A yet another member of the AD susceptibility network, MAP3K2/MEKK2 kinase integrates stress and mitogenic signals to the activation of NF-kB, JNKs, p38, and ERK5 pathways. MAP3K2/MEKK2 directly phosphorylates and activates c-Jun/stress-activated protein kinases (JNKs) and I-kappa-B kinases (IKKs), and promotes motility and invasiveness in cancer cells, in part through control of focal adhesion stability [136, 144]. In other words, MAP3K2 signaling links stress and mitogenic responses, cytoskeletal dynamics, cell adhesions, migration, proto-oncogene signaling, and NF-kB signaling, which are associated with pro-migratory, pro-secretion, pro-inflammatory cellular phenotypes. Altogether, the functional composition of the AD susceptibility network and the specific set of functional interrelations among its constituents – the “functional fingerprint” of the AD susceptibility network – strongly suggest the existence of environmental pressures in AD brains that favor cancer-like, proliferative, pro-migratory phenotypes supported by destabilized cell adhesions/synapses, glycolytic modes of energy production, and, possibly, impaired EMT-like transitions. Chronic hypoxia and/or hypoxia-mimicking conditions in AD brains are examples of the factors that would promote both EMT-like organizational transitions and a switch from oxidative phosphorylation to glycolytic modes of energy generation. As first pointed out by Warburg in the context of cancer etiology, glycolytic modes of energy production, being less efficient than oxidative phosphorylation, are unable to support complex macromolecular organization and thus are bound to lead to cell de-differentiation and tissue degeneration, if/when they become chronically dominant modes of energy generation [16]. Altogether, the emerging AD model appears to account for approximately 60% of proteins comprising the AD susceptibility network, at least conceptually.

**Table 2:** Selected proteins from the AD susceptibility network shown in Figure 8

Name	Description
*Cell-cell and cell-ECM adhesions, cytoskeletal coupling, and the associated cell signaling*	
**ANK-1**	**Ankyrin-1**. Attaches integral membrane proteins to cytoskeletal elements; binds to the cytoskeletal proteins fodrin, tubulin, vimentin, and desmin.
**CDH23**	**Cadherin-23**. Unconventional cadherin. Expressed in the sensory neuroepithelium. Mutated in the Usher syndrome (deafness) and nonsyndromic autosomal recessive deafness DFNB12.
**DSG2**	**CDHF5 / Cadherin family member 5**. Desmosomal cadherin. Involved in the interaction of plaque proteins and intermediate filaments mediating cell-cell adhesion.
**CD2AP**	**CMS / Mesenchyme-to-epithelium transition protein with SH3 domains 1**. An adapter/scaffolding protein connecting membrane proteins and the actin cytoskeleton. In collaboration with CBL-C, modulates the rate of RET turnover and may act as regulatory checkpoint that limits the potency of GDNF on neuronal survival. May play a role in receptor clustering and cytoskeletal polarity in the immunological synapse.
**CD33**	**Putative adhesion molecule**. May act as an inhibitory receptor upon ligand induced tyrosine phosphorylation by recruiting cytoplasmic phosphatase(s) via their SH2 domain(s) that block signal transduction through dephosphorylation of signaling molecules. Induces apoptosis in acute myeloid leukemia (in vitro).
**EPHA1**	**EPH, EPHT, EPHT1 / Ephrin type-A receptor 1**. Receptor tyrosine kinase. Binds promiscuously membrane-bound ephrin-A family ligands residing on adjacent cells, leading to contact-dependent bidirectional signaling into neighboring cells. Upon activation by EFNA1 induces cell attachment to the extracellular matrix, inhibiting cell spreading and motility. Plays a role in angiogenesis and regulates cell proliferation. May play a role in apoptosis.
**APP**	**Amyloid-beta precursor protein**. Functions as a cell surface receptor and performs physiological functions on the surface of neurons related to neurite growth, neuronal adhesion, axonogenesis, and synaptogenesis. Involved in cell mobility and regulation of transcription. Acts as a kinesin I membrane receptor, mediating the axonal transport of beta-secretase and presenilin 1. Involved in copper homeostasis and oxidative stress through copper ion reduction. Can regulate neurite outgrowth through binding to components of the extracellular matrix such as heparin and collagen I and IV.
**Presenilin1 (PSEN1)**	**Catalytic subunit of the gamma-secretase complex,** an endoprotease complex that catalyzes the intramembrane cleavage of integral membrane proteins such as Notch receptors and APP. May play a role in intracellular signaling and gene expression or in linking chromatin to the nuclear membrane. Stimulates cell-cell adhesion though its association with the E-cadherin/catenin complex. Under conditions of calcium influx or apoptosis, cleaves E-cadherin, promoting the disassembly of the E-cadherin/catenin complex and increasing the pool of cytoplasmic beta-catenin.
**SERPINF2**	**A potent and specific plasmin inhibitor**. Inhibits the E-cadherin processing and ensuing dissolution of cell-cell adhesions by the plasminogen activator/plasmin system, thereby inhibiting invasion and metastasis in cancer cells.
*Proliferation-and-migration related signaling*	
**SERPINF1**	**Pigment epithelium-derived factor (PEDF)**. A neurotrophic factor. Promotes differentiation of retinoblastoma and other tumor cells of neuronal origin by promoting neurite outgrowth with concomitant upregulation of neurofilament proteins. Markedly promotes adhesion to collagen-I and inhibits invasiveness. Induces growth arrest and tumor differentiation to a less malignant phenotype. Treatment of endothelial cells with SERPINF1/PEDF significantly increases gamma-secretase activity, which is associated with upregulation of Presenelin 1 and its translocation from the perinuclear region to the plasma membrane. Actions of PEDF are mediated by JNK and p38 MAPK signaling pathways. Activation of the PEDF receptor (PEDFR), which is expressed in many cell types, suppresses NF-kB signaling and expression of pro-migratory genes. Hypoxia is associated with reduced levels of PEDF [134].
**PTK2B**	**FAK2, PYK2, RAFTK / Protein-tyrosine kinase 2-beta / Focal adhesion kinase 2**. Non-receptor protein-tyrosine kinase that regulates reorganization of the actin cytoskeleton, cell polarization, cell migration, adhesion, spreading and bone remodeling. Required for normal macrophage polarization and migration towards sites of inflammation. Regulates cytoskeleton rearrangement and cell spreading in T-cells, and contributes to the regulation of T-cell responses. Functions in signaling downstream of integrin and collagen receptors, immune receptors, G-protein coupled receptors (GPCR), cytokine, chemokine and growth factor receptors, and mediates responses to cellular stress. Regulates numerous signaling pathways. Promotes activation of the phosphatidylinositol 3-kinase (PI3K) and AKT1 signaling cascade as well as the MAP kinase signaling cascade, including activation of MAPK1/ERK2, MAPK3/ERK1 and MAPK8/JNK1. Aberrant PTK2B/PYK2 expression may play a role in cancer cell proliferation, migration and invasion, in tumor formation and metastasis. Elevated PTK2B/PYK2 expression is seen in gliomas, hepatocellular carcinoma, lung cancer and breast cancer.
**INPP5D**	**SHIP-1. Phosphatidylinositol (PtdIns) phosphatase**. Specifically hydrolyzes the 5-phosphate of phosphatidylinositol-3,4,5-trisphosphate (PtdIns(3,4,5)P3) to produce PtdIns(3,4)P2, thereby negatively regulating the PI3K pathways. Acts as a negative regulator of B-cell antigen receptor signaling. Acts as a negative regulator of myeloid cell proliferation/survival and chemotaxis, mast cell degranulation, immune cells homeostasis, integrin alpha-IIb/beta-3 signaling in platelets and JNK signaling in B-cells. Key regulator of neutrophil migration, by governing the formation of the leading edge and polarization required for chemotaxis. Involved in the control of cell-cell junctions, CD32a signaling in neutrophils and modulation of EGF-induced phospholipase C activity. Mediates the activin/TGF-beta-induced apoptosis through its SMAD-dependent expression.
*Ras signaling and migration*	
**RIN3**	**Ras and Rab interactor 3**. A Ras effector and RAB5-directed guanine nucleotide exchange factor (GEF), which is highly expressed and enriched in human mast cells. Stimulation of the receptor tyrosine kinase c-KIT by Stem Cell Factor (SCF) triggers activation of Ras and its downstream effectors. SCF activation of c-KIT is critical for recruiting mast cells to sites of infection or injury. RIN3 functions as an inhibitor of mast cell migration toward SCF, via a mechanism involving c-KIT internalization. RIN1 isoform is highly expressed in mature forebrain neurons and disruption of the RIN1 gene results in elevated LTP and enhanced aversive memories in mutant mice. RIN1 is a regulator of cell adhesion and migration. RIN1 regulates cell migration by coupling endocytosis of activated receptor tyrosine kinases, which is controlled by RAB5 GTPases, and cytoskeletal remodeling, which is controlled by ABL tyrosine kinases [135].
*Stress / mitogenic signaling, NF-kB activation, cytoskeletal dynamics, cell adhesion, and motility*	
**MAP3K2**	**Mitogen-activated protein kinase kinase kinase 2 / MEKK2**. Integrates stress and mitogenic signals to the activation of NF-kB, JNKs, p38, and ERK5 pathways. Directly phosphorylates and activates stress-activated protein kinases (SAPK/JNKs) and I-kappa-B kinases (IKKs). Promotes motility and invasiveness in cancer cells, in part through control of focal adhesion stability [136].
*Regulation of glycolysis*	
**PFKFB2**	**6-phosphofructo-2-kinase/fructose-2,6-bisphosphatase 2**. PFKFB (isoforms PFKFB1-4) sets the pace of glycolysis in cells by controlling intracellular levels of fructose 2,6-bisphosphate (F2,6BP). F2,6BP is the most potent allosteric activator of PFK-1 (6-phosphofructo-1-kinase), which catalyzes the essentially irreversible, commitment step in the glycolytic pathway. F2,6BP allows PFK-1 to bypass the inhibition by ATP, thereby permitting cells to maintain high rates of glycolysis even at physiological ATP concentrations. Induced by hypoxia, hypoxia mimics, growth factors, and hormones. Overexpressed in a large variety of cancers. Required for cell transformation by oncogenic Ras [137].
*Regulation of oxidative phosphorylation*	
**MEF2C**	**Myocyte-specific enhancer factor 2C**. Transcriptional factor MEF2 (isoforms MEF2A/B/C/D). MEF2A/C/D isoforms are highly expressed in the brain, where MEF2s regulate neuronal development, synaptic plasticity, activity-dependent survival, learning and memory. MEF2C facilitates learning and memory by negative regulation of synapse numbers and function. In stimulated mast cells, MEF2C isoform regulates c-JUN expression. MEF2D isoform was shown to act as a mitochondrial transcription factor that regulates expression of the mtDNA-encoded NADH dehydrogenase 6 (ND6), an essential component of complex I, whose insufficiency leads to a severe disruption of complex I structure and impairment of oxidative phosphorylation. MEF2D and ND6 protein levels are greatly reduced in brain mitochondria of human Parkinson's disease (PD) patients and MPTP-treated mice modeling PD. An average 50% reduction of the mtDNA ND6 transcripts has been also detected in AD brains. Ablation of MEF2A isoform in mice disrupts mitochondrial and cyto-architectural integrity in the post-natal heart, leading to sudden death [138]. MEF2 mediates TGF-beta-induced EMT in hepatocellular carcinoma, promoting migration and invasion of cancer cells. MEF2 synergizes with activated Notch in Drosophila to promote hyperproliferative and invasive cellular phenotypes via JNK activation.
*Inflammation*	
**TREM1**	**Triggering receptor expressed on myeloid cells 1 (ACUTE INFLAMMATION)**Stimulates neutrophil- and monocyte-mediated inflammatory responses. Triggers release of pro-inflammatory chemokines and cytokines, as well as increased surface expression of cell activation markers. Amplifier of inflammatory responses triggered by bacterial and fungal infections and is a crucial mediator of septic shock. Strongly expressed in acute inflammatory lesions caused by bacteria and fungi.
**TREM2**	**Triggering receptor expressed on myeloid cells 2 (CHRONIC INFLAMMATION)**May have a role in chronic inflammation, stimulating production of constitutive rather than inflammatory chemokines and cytokines. Triggers activation of the immune responses in macrophages and dendritic cells. Mutated in polycystic lipomembranous osteodysplasia with sclerosing leukoencephalopathy (PLOSL). PLOSL is a recessively inherited disease characterized by a combination of psychotic symptoms rapidly progressing to presenile dementia and bone cysts restricted to wrists and ankles.

In all likelihood, the remaining proteins can be also assimilated within the same model/framework, promoting its further evolution and refinement. For example, in regard to HLA-DRB1 and HLA-DRB5, recent work suggests that the establishment of appropriate connections between neurons in the developing CNS is controlled by a balance between synaptogenic molecules and proteins that negatively regulate synapse formation and plasticity. Surprisingly, many of these newly identified synapse-limiting molecules are classic “immune” proteins. In particular, major histocompatibility complex class I (MHCI) molecules regulate neurite outgrowth, the establishment and function of cortical connections, activity-dependent refinement in the visual system, and long-term and homeostatic plasticity [145]. MHCI proteins appear to function as co-receptors in cell-cell interactions. However, their mechanism of action in the context of neuronal interactions is largely unknown. According to the UniProt database description, DIP2A may play a role in development, by providing positional cues for axon pathfinding and patterning in the central nervous system. DIP2A was recently identified as a receptor for FLST1 (follistatin-like 1), an extracellular secreted glycoprotein, which interacts with the TGF-beta superfamily of proteins, such as activin, TGF-beta, and BMP2/4, and modifies their interactions with cognate receptors, but whose physiological function is unclear. It is worth noting that the FLST5 isoform is the 3^rd^-degree APP interactor (APP-X11-CX40.1-FLST5), which was not included in the present analysis due to its relatively low score of interaction probability. BIN1/Bridging Integrator I/Amphiphysin II, which interacts with RIN3 and dynamin-1, is a key component of endocytic machinery that mediates such cell-environmental interactions as receptor-mediated endocytosis, migration, and cell-cell and cell-ECM interactions. The cholesterol/lipid metabolism in the brain is largely separated from the rest of the body by the blood-brain barrier. At the same time, cholesterol and phospholipids constitute critical energy/matter resources for neurons, especially during active remodeling or outgrowth. ApoE is the major apolipoprotein that mediates transport of cholesterol-rich lipoproteins in the brain. Another abundant apolipoprotein in the brain is ApoJ (also known as clusterin (CLU)); ApoE and ApoJ are present on distinct populations of lipoprotein particles. ApoE and its receptors, such as LRP1, ABCA1, SORL1, and APP, mediate the binding/adhesion of circulating and ECM-deposited cholesterol/lipoproteins to cells, followed by lipoprotein internalization and metabolism. Therefore, apolipoproteins and their receptors play a key role in cholesterol/lipid redistribution among different cells and cell types in the brain, and thus define their migration and metabolic preferences and modes of functioning [146, 147]. CLU/Clusterin/Apolipoprotein-J is a secreted glycoprotein, which interacts both with secreted ligands, such as TGF-beta, EGF, SERPINF2/PEDF, fibronectin, and with cell surface receptors, such as TGF-beta receptors, APP and LRP2. It is expressed at low level in most cells, but is markedly upregulated upon a great variety of stresses. In addition to its role in lipoprotein transport and metabolism, CLU/ApoJ also acts as a stress-activated extracellular chaperone that binds exposed hydrophobic parts of partially unfolded proteins, preventing their aggregation. Binding to cell surface receptors triggers receptor-mediated endocytosis of the chaperone-client complex and its subsequent lysosomal or proteasomal digestion. Intracellular CLU/ApoJ enhances NF-kB activity by promoting proteasomal degradation of its inhibitor I-kappaB. CLU/ApoJ overexpression is thought to be a nonspecific cytoprotective response to a great variety of insults, which promotes stress-induced migration and invasiveness. Consequently, CLU/ApoJ is one of the major targets in cancer therapy, as it is often overexpressed in cancer cells in response to cytotoxic agents, conferring resistance to therapy [148].

In terms of refining the evolving AD model, it becomes possible to hypothesize that ApoE, CLU/ApoJ, and most likely other secreted trophic factors (e.g., sAPPalpha) may function as molecular “scouts/scavengers” that are secreted by cells into their immediate exracellular environments to procure energy/matter resources, such as proteins, lipids, lipoproteins, cholesterol, glycoproteins, carbohydrates, and nucleic acids, and to deliver them to cells expressing appropriate receptors for internalization and digestion, acting in an autocrine/paracrine signaling manner and thus directing cellular migration and/or outgrowth toward resource-rich environments. This type of resource procurement is expected to be especially important and effective in disorganized tissue contexts such as wounds, sites of bacterial and productive and/or reactivated viral infections, sites of inflammation, and in regeneration and development. Indeed, upon experimentally induced nerve injury in rats, ApoE is produced and accumulates extracellularly at the site of injury to levels 100- to 200-fold higher than normal, comprising up to 5% of total soluble protein in regenerating nerve segment. Immediately after injury, resident macrophages are activated and begin producing and secreting ApoE to scavenge and gobble up cholesterol and lipids released at the site of injury. The secreted ApoE captures lipids and cholesterol from the environment and delivers them back to macrophages, where they are stored. Monocytes invading the wound rapidly differentiate into macrophages and also begin producing and secreting ApoE to procure “spilled” lipids and cholesterol [146]. Metal ions, such as copper, zinc, and iron, are an example of another essential resource that can be procured from extracellular environment in an analogous manner, with the help of secreted molecules that bind metal ions, such as ferritin and perhaps sAPPalpha and A-beta, which are known to efficiently chelate metal ions. As a healing, regenerating, or developing tissue becomes progressively organized and stabilized, via the formation of stable cell-cell adhesions/junctions/synapses, cell differentiation, and the MET-like, organizational transitions toward stable, large-scale, cytoskeletal architectures, the largely random, undifferentiated, and diffusion-driven procurement and exchange of energy/matter resources would give way to a differentiated, organized, and thus more efficient system of intercellular exchanges.

The outlined scenario is consistent with a large variety of experimental observations pertaining to cell/tissue physiology, in general, and to AD, in particular. As an example, it may help to explain the paradoxical involvement of many seemingly odd proteins in AD pathogenesis, such as C-reactive protein [149], plasmin/plasminogen system [150, 151], complement proteins [152, 153], alpha-2-macroglobulin [152], ferritin [154, 155], fibrinogen [156], ceruloplasmin [157], haptoglobin [158], hepcidin [159], mannose-binding lectin [160], and serpins (e.g., SERPINF2/alpha-2-antiplasmin, neuroserpin and alpha(1)-antichymotrypsin) [151, 161]. All these proteins are acute-phase response proteins that are secreted by cells and organs (e.g., the liver) upon injury. They constitute a part of the innate immune system and act to “clear” sites of injury and inflammation by helping surviving cells to digest and absorb cellular debris, ECM, dying and damaged cells, and invaders, such as bacteria, eventually leading to wound healing, regeneration, and the restoration of functional tissue architecture. Two other proteins in the list of acute-response proteins are serum amyloid A (an apolipoprotein associated with high-density lipoproteins (HDLs)) and serum amyloid P component, which form amyloid deposits in pathological conditions and which gave name to the term “amyloidosis”. Both proteins are highly conserved in all animals, suggesting their critical role in supporting multi-cellularity. Given the separation of the brain's cholesterol/lipid metabolism from the rest of the body, a critical role of cholesterol for neuronal functions, and a high abundance of cholesterol in the brain (estimated 25% of total body cholesterol), ApoE and CLU/ApoJ may thus function as the brain-specific counterparts of injury/damage response, potentially explaining why certain genetic variants of these apolipoproteins are associated with the AD risk. Moreover, as a recent study shows, ApoE4 isoform, unlike other ApoE isoforms, activates NF-kB signaling and downregulates SirT1 in cultured neurons [162], suggesting that neurons expressing ApoE4 isoform may have a significantly lower threshold in switching between alternative modes of functioning, perhaps making the neuronal networks of ApoE4-carriers more plastic in the younger age at the expense of their lower stability in the old age. Acute-response proteins also promote vascular permeability, thus providing a potential explanation for the compromised blood-brain barrier in AD, which, to a large extent, relies on tight junctions between adjacent barrier cells.

Summarizing, even a brief investigation guided by the evolving AD model reveals a large and diverse body of experimental evidence suggesting the existence of environmental pressures that favor cancer-like cellular phenotypes in AD brains. In addition, the analysis reveals that the stabilization of these phenotypes is likely achieved via acquisition of somatic mutations and/or epigenetic modifications that target key molecules, pathways, and processes that are essential for maintenance of cancer-like cellular phenotypes. Once stabilized, cancer-like cellular phenotypes are expected to constitute and promote microenvironments that support their own survival and prosperity at the expense of collective organization.

To conclude with therapeutic implications, consider the following plausible scenarios of AD pathogenesis. In the first scenario, an inherited or acquired genetic or epigenetic “mutation” in presenilin 1 may result in unstable adhesions/synapses (perhaps providing the advantage of enhanced plasticity early in life at the expense of lower stability in the old age). Over time, increasingly unstable adhesions/synapses would lead to an increased production of A-beta and oxidative stress, which in turn would create sustained environmental pressures favoring (glyco)lytic modes of energy generation and selecting those cells that have acquired genetic and/or epigenetic “mutations” stabilizing (glyco)lytic metabolism and the corresponding cellular phenotypes, thus leading to progressive tissue degeneration. In the second scenario, a chronic brain hypoxia (which can have a variety of different causes, including vascular disorders, hypoperfusion, impaired oxygen-carrying capacity of blood, chemical hypoxia due to environmental pollutants, sedentary lifestyles, inadequate dietary habits, and combinations thereof) may create sustained environmental pressures favoring (glyco)lytic modes of energy production and selecting those cells that have acquired genetic or epigenetic alterations stabilizing (glyco)lytic metabolism. In turn, the stabilization of (glyco)lytic metabolism would lead to destabilization of adhesions/synapses, thus leading to an increased production of A-beta and oxidative stress and so forth. In the third scenario, impaired one-carbon metabolism (which can have a variety of different causes, such as vitamin B and/or folate deficiencies, chronic methylmercury poisoning, and/or genetic or epigenetic “mutations” affecting certain metabolic enzymes) may alter or impair mitochondrial function, thus destabilizing adhesions/synapses and leading to an increased A-beta production and oxidative stress and so forth. Moreover, altered or impaired mitochondrial function can be a consequence of chronic inflammation and oxidative stress, but it can be also a cause of chronic inflammation and oxidative stress. Altered or impaired mitochondrial function can be a consequence of impaired one-carbon metabolism, but it can be also a cause of deficient one-carbon metabolism. In turn, deficient one-carbon metabolism can alter the methylation status of the presenilin 1 gene, destabilizing adhesions/synapses and so forth, going on in circles within circles within circles, a typical signature of a multi-scale network. Furthermore, in addition to many “entrances” into the AD network, various plausible scenarios of the AD network evolution, i.e., specific evolutionary trajectories of AD pathogenesis, are not mutually exclusive and some or many of them may turn out to be correct, being realized in different AD patients or in different classes of AD patients. The same or similar considerations may also apply to cancer and other degenerative disorders. In short, the familiar, unidirectional logic of cause and effect may not be sufficient for analyzing dynamic networked processes and their interdependent co-evolution toward persistent imbalances and locked-in modes/states that appear to underlie the pathogenesis of complex diseases, such as cancer and Alzheimer's disease.

The emerging AD model suggests that AD pathogenesis involves a life-long evolution of a multi-scale network comprising inter-molecular, inter-cellular, inter-organ, and organism-environment interactions. In the course of its evolution, the AD network gradually acquires a number of “defects” that eventually lock the network in a pathological, degenerative mode of functioning. According to this view, the underlying cause of AD is not a failure of a particular molecule, pathway, structure, or process but a failure of a dynamic network of interdependent and interacting molecules, pathways, and processes. Therefore, it may be more productive to think in terms of developing an AD therapeutic system that addresses key “defects” in the multi-scale AD network, rather then in terms of a single drug targeting this molecule or that pathway in the AD “mechanism”, which simply does not seem to exist, in the familiar sense of a linear, cause-and-effect chain. The AD therapeutic system may need to be paired with an adequate diagnostic system and to be flexible enough to allow for individualized treatment regimes. Different AD patients may have different “origins/roots” of their disease that feed into a few common “branches”, such as chronic inflammation, oxidative stress, and altered metabolism, which in turn fuel the “trunk” of the A-beta cascade that promotes AD blossoming later in life.

## MATERIALS AND METHODS

### A core model of Alzheimer's disease

A model of Alzheimer's disease that was recently put forward by one of the authors (D.E.B.) [1-4] was used as a core model to guide the search and analysis of AD-relevant research data and knowledge by an ISKRA algorithm. Simplifying, the model posits that neurodegenerative processes in Alzheimer's disease are caused by dysregulation of the developmentally-related, physiological mechanisms that balance neurite extension and synaptogenesis versus neurite retraction and synaptic reorganization. The two opposing physiological processes are postulated to account for neuronal plasticity, which is required during development and is maintained in long-lived species throughout life to enable continuous adaptation to changing environments via such processes as learning, memory, and cognition. In this model, amyloid-beta precursor protein (APP) plays the role of a principal “plasticity switch”, in the sense that APP is postulated to integrate extracellular and intracellular signals to enable a switch between the retraction-and-reorganization and outgrowth-and-(re)connection modes in neuron/neurite functioning. Mechanistically, trophic factors, such as neurotrophins, growth factors, chemoattractants, hormones, and axon-guidance factors, act via APP-associated signaling complexes to promote neurite proliferation, migration, and synaptogenesis, whereas anti-trophic factors, such as neurotrophic factor withdrawal, chemorepellents, stress/insult, and certain cytokines, act to promote neurite retraction and synaptic reorganization. The concomitant processing of APP by alpha-, beta-, and gamma-secretases leads to the generation of two classes of APP fragments that promote two counter-opposing processes [5]. One class of APP proteolytic fragments, which includes sAPP-alpha and alpha-CTF, supports neurite extension and inhibits neuronal cell death [6-8], whereas other class of APP fragments, which includes amyloid-beta (A-beta), sAPP-beta, J_CASP_, and C31, promote neurite/synapse collapse/retraction and neuronal cell death [9-11]. The APP proteolytic fragments generated by secretases are postulated to amplify the rate and/or extent of two counter-opposing processes, disconnection-and-retraction and proliferation-and-reconnection. Overall, the model presents Alzheimer's disease as a trophic versus anti-trophic signaling imbalance, which is mediated by APP-associated signaling complexes and amplified by APP fragments generated in the course of APP signaling and/or metabolism. In addition, the model draws conceptual parallels between Alzheimer's disease and the emergence and progression of the neoplastic phenotype in cancer. Whereas in cancer, the Darwinian-like selection operates at the scale of cells, leading to the acquisition and retention of mutations that support and promote uncontrolled proliferation and migration, in neurodegeneration, the Darwinian-like selection is proposed to operate at the molecular scale, leading to a progressive acquisition of degenerative cellular phenotypes, due to progressive dysregulation of the APP-associated signaling network in affected neurons. The model therefore assumes a key role of the APP-associated signaling network in the pathogenesis of Alzheimer's disease [1, 2].

### The APP protein interaction network as a guiding dataset

A dataset of proteins comprising the APP-associated protein interaction network (APP connectome) was assembled in the following manner. Previously, we used a system-scale screening to identify 46 putative interacting partners of X11/Mint1 protein, one of the three major scaffolding proteins associated with APP [12]. Strategically positioned at cross-roads of signaling pathways, scaffolding proteins often function as hubs that integrate and coordinate various aspects of cellular physiology, allowing the cell to perform as a dynamic, coherent whole. The proteins interacting with X11/Mint1 were treated as 2^nd^-degree APP interactors. To these we added proteins that interact with Fe65 and JIP1, two other major scaffolds associated with APP, plus the 1^st^-degree APP interactors reported in research literature and databases. APP connectome was used to drive a self-guiding evolution of the core model of Alzheimer's disease, by employing both the model and APP connectome as “guides” and “filters” in the directed search and analysis of research literature and databases. Because one of the main purposes of the model evolution is self-consistent assimilation and integration of data, the dataset of APP interactors was kept open, in the sense that the 3^rd^- and higher-degree APP interactors were included in the analysis and assimilation of data as needed.

## SUPPLEMENTARY MATERIAL TABLE


