# Supplementary material for: Dynamic self-guiding analysis of Alzheimer's disease
Source: Oncotarget. 2015 May 20;6(16):14092–122. doi: 10.18632/oncotarget.4221 (PMC4546454; doi:10.18632/oncotarget.4221)
Supplement: Supplementary file 1 [file oncotarget-06-14092-s001.pdf]

Dynamic self-guiding analysis of alzheimer's disease

Supplementary Material

Table 1. APP functional connectome. Descriptions of selected proteins comprising the branch of APP connectome that couples and potentially coordinates cell adhesions/synapses, adhesions-to-nucleus signaling, transcriptional responses, stress-activated signaling, inflammation/immune responses, and proliferation and migration.

Name	Description	Degree
Establishment, maintenance, and remodeling of adhesions/synapses; adhesions-to-nucleus signaling		
**APP**	**Amyloid-beta precursor protein.** Functions as a cell surface receptor and performs physiological functions on the surface of neurons related to neurite growth, neuronal adhesion, axonogenesis, and synaptogenesis. Involved in cell mobility and regulation of transcription. Acts as a kinesin I membrane receptor, mediating the axonal transport of beta-secretase and presenilin 1. Involved in copper homeostasis and oxidative stress through copper ion reduction. Can regulate neurite outgrowth through binding to components of the extracellular matrix such as heparin and collagen I and IV.	self
**Presenilin 1**	**Catalytic subunit of the gamma-secretase complex,** an endoprotease complex that catalyzes the intramembrane cleavage of integral membrane proteins such as Notch receptors and APP. May play a role in intracellular signaling and gene expression or in linking chromatin to the nuclear membrane. Stimulates cell-cell adhesion through its association with the E-cadherin/catenin complex. Under conditions of calcium influx or apoptosis, cleaves E-cadherin promoting the disassembly of the E-cadherin/catenin complex and increasing the pool of cytoplasmic beta-catenin	1st
Scaffolding of adhesions, synapses, and coupling to the cytoskeleton		
**X11 (Mint1)**	**A major APP-associated scaffold.** Together with CASK and Veli forms a tripartite complex that acts as a nucleation site for the assembly of proteins involved in synaptic junctions and synaptic vesicle exocytosis. The X11/CASK/Veli complex plays a role in establishing and maintaining the asymmetric distribution of channels and receptors at the plasma membrane of polarized cells.	1st
**CASK**	**Calcium/calmodulin-dependent serine protein kinase** (MAGUK family). Mutlidomain scaffold involved in synaptic transmembrane protein anchoring and ion channel trafficking. Binds to cell-surface proteins, including neurexins and syndecans. May mediate a link between the extracellular matrix and the actin cytoskeleton via its interaction with syndecans and with the actin/spectrin-binding protein 4.1.	2nd
**SHANK1**	**SH3 and multiple ankyrin repeat domains 1**. An adapter protein in the postsynaptic density (PSD) of excitatory synapses that interconnects receptors of the postsynaptic membrane including NMDA-type and metabotropic glutamate receptors via complexes with GKAP/PSD-95 and Homer, respectively, and the actin-based cytoskeleton. May play a role in the structural and functional organization of the dendritic spine and synaptic junction.	3rd
Specialized cell-cell junctions/synapses		
**GJD4 (CX40.1)**	**Gap junction delta-4 protein.** A member of the connexins family of proteins that form gap junctions, the most common type of electrical synapses. Gap junctions and connexins are involved in synchronization of neuronal firing. The X-linked form of Charcot–Marie–Tooth disease (CMT), an inheritable progressive neuropathy, is caused by more then 400 different mutations in the GJB1 gene.	2nd
**TJP1 (ZO-1)**	**Tight junction scaffold ZO-1.** Organizes and couples adhesion, cytoskeletal, and signaling proteins, and transcription factors. Plays a role in regulation of cell migration by targeting CDC42BPB to the leading edge of migrating cells. Involved in neuronal dendrite morphogenesis by mediating and stabilizing the transient filopodia-filopodia contacts/adhesions in developing dendrites. Interacts with connexins and may play a scaffolding role in gap junctions.	3rd
**TJP2 (ZO-2)**	**Tight junction and adherens junction scaffold ZO-2.** Organizes and couples adhesion, cytoskeletal, and signaling proteins, and transcription factors. Interacts with connexins and may play a scaffolding role in gap junctions.	3rd

Recognition and coupling of extracellular environment to the cytoskeleton and intracellular signaling		
**LRP1**	**Prolow-density lipoprotein receptor-related protein 1**. Endocytic receptor involved in endocytosis and in phagocytosis of apoptotic cells. Required for early embryonic development. Involved in cellular lipid homeostasis. Involved in the plasma clearance of chylomicron remnants and activated LRPAP1 (alpha 2-macroglobulin), as well as the local metabolism of complexes between plasminogen activators and their endogenous inhibitors. Recognizes more than 30 distinct ligands, binds a large number of adaptor proteins in a phosphorylation-specific manner, associates with and modulates activity of other transmembranes receptors, such as integrins and receptor tyrosine kinases. Plays a role in integrin maturation and recycling, focal adhesion disassembly, regulation of cell migration, and modulation of the integrity of the blood-brain barrier.	2nd
**CASPR3/4**	**Cell recognition molecule of the neurexin family.** Caspr3 is detected along axons in the corpus callosum, spinal cord, basket cells in the cerebellum and in peripheral nerves, as well as in oligodendrocytes. Expression of Caspr4 is more restricted to specific neuronal subpopulations in the olfactory bulb, hippocampus, deep cerebellar nuclei, and the substantia nigra. Similar to the neurexins, the cytoplasmic tails of Caspr3 and Caspr4 interacts differentially with PDZ domain-containing proteins of the X11/CASK/Veli complex.	2nd
**CDH1**	**Cadherin-1/E-cadherin.** Cadherins are calcium-dependent cell adhesion proteins. They preferentially interact with themselves in a homophilic manner, mediating cell recognition and adhesion; cadherins may thus contribute to the sorting of heterogeneous cell types. E-cadherin is involved in mechanisms regulating cell-cell adhesions, mobility and proliferation of epithelial cells. E-cadherin is a tumor suppressor that mediates contact inhibition of cell proliferation and inhibits invasion and metastasis in a variety of contexts. It is a ligand for integrin alpha-E/beta-7.	3rd
**CDH2**	**Cadherin-2/N-cadherin.** Mediates cell migration, axonal guidance, and synaptic plasticity in the nervous system. The cytoplasmic domain of N-cadherin interacts with beta-catenin and forms a structural adaptor that links to the cytoskeleton. N-cadherin mediates adhesion at neuronal synapses, linking synaptic activity and synaptic plasticity. Involved in LTP and learning and memory. N-cadherin is essential for cortical and other neural tissues morphogenesis, and blocking or deleting N-cadherin causes disruption of ordered cell architectures, in part by disrupting the adherens junctions in neuroepithelial and/or glial cells.	3rd
**CTNNB1**	**Catenin beta-1.** Key downstream component of the canonical Wnt signaling pathway. Acts as a coactivator for transcription factors of the TCF/LEF family, leading to activate Wnt responsive genes. Involved in the regulation of cell adhesion. Acts as a negative regulator of centrosome cohesion. Involved in the CDK2/PTPN6/CTNNB1/CEACAM1 pathway of insulin internalization. Blocks anoikis of malignant kidney and intestinal epithelial cells and promotes their anchorage-independent growth by down-regulating DAPK2.	2nd
**SDC1**	**Syndecan-1.** Cell surface proteoglycan that bears both heparan sulfate and chondroitin sulfate and that links the cytoskeleton to the interstitial matrix.	3rd
**SDC2**	**Syndecan-2.** Cell surface proteoglycan that bears heparan sulfate. Regulates dendritic arbor morphogenesis.	3rd
**SDC3**	**Syndecan-3.** Cell surface proteoglycan that may bear heparan sulfate. May have a role in the organization of cell shape by affecting the actin cytoskeleton, possibly by transferring signals from the cell surface in a sugar-dependent mechanism.	3rd
**VCAN**	**Versican.** May function in cell recognition, possibly by connecting extracellular matrix components and cell surface glycoproteins. May play a role in intercellular signaling and in connecting cells with the extracellular matrix. May take part in the regulation of cell motility, growth and differentiation. Binds hyaluronic acid.	3rd
**NCAN**	**Neurocan** core protein. Chondroitin sulfate proteoglycan. May modulate neuronal adhesion and neurite growth during development by binding to neural cell adhesion molecules (NG-CAM and N-CAM). Binds to hyaluronic acid.	3rd
**BCAN**	**Brevican** core protein. May play a role in the terminally differentiating and adult nervous system during postnatal development. Could stabilize interactions between hyaluronan (HA) and brain proteoglycans.	3rd
**ITGA1**	**Integrin alpha-1/beta-1** is a receptor for laminin and collagen. It recognizes the proline-hydroxylated sequence G-F-P-G-E-R in collagen. Involved in anchorage-dependent, negative regulation of EGF-stimulated cell growth.	3rd
**ITGA2**	**Integrin alpha-2/beta-1** is a receptor for laminin, collagen, collagen C-propeptides, fibronectin and E-cadherin. It recognizes the proline-hydroxylated sequence G-F-P-G-E-R in collagen. It is responsible for adhesion of platelets and other cells to collagens, modulation of collagen and collagenase gene expression, force generation and organization of newly synthesized extracellular matrix.	3rd
**ITGA5**	**Integrin alpha-5/beta-1** is a receptor for fibronectin and fibrinogen. It recognizes the sequence R-G-D in its ligands.	3rd
Calcium signaling		
**CACNA1B**	**Voltage-dependent N-type calcium channel subunit alpha-1B.** Voltage-sensitive calcium channels (VSCC) mediate the entry of calcium ions into excitable cells and are also involved in a variety of calcium-dependent processes, including hormone or neurotransmitter release, gene expression, cell motility, cell division and cell death. Calcium channels containing alpha-1B subunit may play a role in directed migration of immature neurons.	2nd
Migration: adhesion, ECM remodeling, and cytoskeletal dynamics		
**CSPG4**	**Chondroitin sulfate proteoglycan 4.** Plays a role in cell proliferation and migration. May also inhibit neurite outgrowth and growth cone collapse during axon regeneration. Cell-surface receptor for collagen alpha 2(VI), which may confer cells ability to migrate on that substrate. Binds through its extracellular N-terminus growth factors, extracellular matrix proteases modulating their activity. May regulate MMP16-dependent degradation and invasion of type I collagen. Functions also as a signal transducing protein by binding through its cytoplasmic C-terminus scaffolding and signaling proteins. May stimulate alpha-4, beta-1 integrin-mediated adhesion and spreading.	2nd
**MMP15**	**Matrix metalloproteinase-15.** Endopeptidase that degrades various components of the extracellular matrix. Upregulated in breast cancer cells upon the transition from a pre-invasive to an invasive phenotype. Matrix metalloproteinases are an essential functional component required for cellular migration and invasion, and for tissue remodeling and restoration.	2nd
**TIMPs**	**Metalloproteinase inhibitors** (TIMP1,2,3,4). TIMPs exhibit differential activities toward different MMPs and ADAMs. Involved in integrin signaling. May form part of a tissue-specific acute response to remodeling stimuli. Involved in cell differentiation, migration, and cell death. TIMPs promote fibroblast adhesion through stabilization of focal adhesion contacts, which is correlated with an increase in N-cadherin expression at the cell surface.	3rd
**RELN**	**Reelin.** Extracellular matrix serine protease that plays a role in layering of neurons in the cerebral cortex and cerebellum. Regulates microtubule function in neurons and neuronal migration. Affects migration of sympathetic preganglionic neurons in the spinal cord, where it seems to act as a barrier to neuronal migration. Enzymatic activity is important for the modulation of cell adhesion.	2nd
**ENAH**	**Ena/VASP proteins** are actin-associated proteins involved in a range of processes dependent on cytoskeleton remodeling and cell polarity such as axon guidance and lamellipodial and filopodial dynamics in migrating cells. ENAH induces the formation of F-actin rich outgrowths in fibroblasts.	2nd
Proliferation and migration: pathfinding and autocrine and paracrine signaling		
**UNC5D**	**Netrin receptor UNC5D.** Receptor for netrin. May be involved in axon guidance by mediating axon repulsion of neuronal growth cones in the developing nervous system upon ligand binding. Axon repulsion in growth cones may be mediated by its association with DCC. Acts as a dependence receptor, inducing apoptosis when not associated with netrin.	3rd

**CXCR2**	**C-X-C chemokine receptor type 2.** A GPCR for the CXCL7/PPBP chemokine and interleukin-8. Binding of IL-8 to the receptor leads to activation of neutrophils. This response is mediated via a G-protein that activates a phosphatidylinositol-calcium second messenger system. CXCR2 is the closest homologue to Kaposi's sarcoma herpesvirus-G protein-coupled receptor (KSHV-GPCR), which is known to be constitutively activated and able to cause oncogenic transformation. CXCR2 is aberrantly expressed in many tumors, where it exerts potent proliferative, pro-survival, and pro-migratory effects.	3rd
**CXCL7 (PPBP)**	**Platelet basic protein.** A cytokine, growth factor, and chemoattractant. A ligand for CXCR1 and CXCR2. Stimulates DNA synthesis, mitosis, glycolysis, intracellular cAMP accumulation, prostaglandin E2 secretion, and synthesis of hyaluronic acid and sulfated glycosaminoglycan. Stimulates the formation and secretion of plasminogen activator by human synovial cells.	3rd
**CXCR4**	**C-X-C chemokine receptor type 4.** A GPCR for the CXCL12/SDF-1 chemokine that transduces a signal by increasing intracellular calcium ion levels and enhancing MAPK1/MAPK3 activation. Plays an essential role in developmental morphogenesis. CXCL12/CXCR4 signaling controls axon morphogenesis and directs neuronal migration and axonal pathfinding in the developing nervous system. In the adult brain, CXCL12 is thought to influence neurogenesis as well as recruitment of brain resident and non-resident circulating cells toward sites of lesion. Together with CXCR2, CXCR4 is among the most promising GPCR targets in anti-cancer drug development, as it is aberrantly expressed in many tumors, where it exerts potent proliferative, pro-survival, and pro-migratory effects. A major co-receptor for HIV-1.	3rd
**CXCL12 (SDF-1)**	**Stromal cell-derived factor 1.** Activates the CXCR4 chemokine receptor to induce a rapid and transient rise in the level of intracellular calcium ions and chemotaxis. Acts as a positive regulator of monocyte migration and a negative regulator of monocyte adhesion via the LYN kinase. CXCL12/CXCR4 signaling plays an essential role in the development of the nervous system and other tissues.	3rd
**CCR5**	**C-C chemokine receptor type 5.** A GPCR for a number of inflammatory CC-chemokines including MIP-1-alpha, MIP-1-beta and RANTES and subsequently transduces a signal by increasing intracellular calcium levels. May play a role in the control of granulocytic lineage proliferation or differentiation. A major co-receptor for HIV-1.	3rd
Stress-activated signaling		
**JIP1**	**A major APP-associated scaffold.** Assembles specific components of the MAPK cascade to form a functional JNK signaling module. Required for JNK activation in response to excitotoxic stress. May also participate in ApoER2-specific reelin signaling. Directly, or indirectly, regulates GLUT2 gene expression and beta-cell function. Appears to have a role in cell signaling in mature and developing nerve terminals.	1st
**JNK**	**Stress-activated protein kinase/c-Jun N-terminal kinase (SAPK/JNK).** Involved in cell proliferation, differentiation, migration, transformation, and programmed cell death. Promotes stressed cell apoptosis and starvation-induced induction of autophagy. Phosphorylates AP-1 components, including c-JUN. Other substrates include neurofilament heavy chain, MAP1B, YAP1, and SirT1. Regulates microtubule dynamics, controlling neurite elongation in cortical neurons.	2nd
**MAP2K7**	**JNK-activating kinase 2/MKK7.** Dual specificity protein kinase. Acts as an essential component of the stress-activated protein kinase/c-Jun N-terminal kinase (SAP/JNK) signaling pathway. Directly activates JNK. Has a specific role in JNK signaling activated by pro-inflammatory cytokines. Together with DLK and JNK1, MKK7 regulates microtubule bundling leading to neurite elongation via MAP1B phosphorylation. MKK7-mediated regulation of JNK is uniquely critical for both axon elongation and radial migration in the developing brain. MKK7 is activated by MAP3K1/MEKK1, MAP3K2/MEKK2, and MAP3K3.	2nd
**VRK2**	**Ser/Thr protein kinase** that regulates several signal transduction pathways. Modulates the stress response to hypoxia and cytokines, and this is dependent on its interaction with JIP1, which assembles mitogen-activated protein kinase (MAPK) complexes. Downregulates the transactivation of transcription induced by ERBB2, HRAS, BRAF, and MEK1.	2nd
Scaffolding of transcription and DNA double-strand break repair complexes		
**Fe65**	**A major APP-associated scaffold.** Scaffolds transcriptionally active APP/AICD complexes and the APP/AICD/Fe65/Tip60 complexes involved in DNA double-strand break repair. Required for histone H4 acetylation at double-strand breaks (DSBs). Binds to phosphorylated histone H2AX at DSBs and recruits JNK1/MAPK8 to DSBs. Its ability to specifically bind modified histones and chromatin-modifying enzymes such as KAT5/Tip60 may explain its trancription activation activity.	1st
Histone acetylation, chromatin remodeling, and regulation of transcription		
**KAT5 (Tip60)**	**Histone acetyltransferase.** A transcriptional co-activator and a key constituent of the APP/AICD/Fe65/Tip60 machinery. Catalytic subunit of the NuA4 histone acetyltransferase complex, which is involved in transcriptional activation of select genes, principally via acetylation of nucleosomal histones H4 and H2A. This complex may be required for the activation of transcriptional programs associated with oncogene and proto-oncogene mediated growth induction. KAT5/Tip60 is a tumor suppressor and a frequent target of monoallelic loss in human carcinomas.	2nd
**CBP (CREBBP)**	**CREB-binding protein.** Acetylates histones, giving a specific tag for transcriptional activation. Also acetylates non-histone proteins, like NCOA3 and FOXO1. Binds specifically to phosphorylated CREB and enhances its transcriptional activity toward cAMP-responsive genes. A tumor suppressor. Mutated in relapsed acute lymphoblastic leukaemia (ALL).	3rd
**p300**	**Histone acetyltransferase.** Regulates transcription via chromatin remodeling. Acetylates all four core histones in nucleosomes as well as other nonhistone targets. Binds phosphorylated CREB to regulate cAMP-responsive genes. A tumor suppressor. Defects in p300 may play a role in epithelial cancers. Chromosomal aberrations involving p300 may be a cause of acute myeloid leukemias. Mutated in Rubinstein-Taybi syndrome 2, a disorder characterized by craniofacial abnormalities, postnatal growth deficiency, broad thumbs, broad big toes, mental retardation and a propensity for development of malignancies. Interacts with SMADs, NF-kB, CREB, HIF1, TP53, Sp1, NCOA3, and estrogen receptor.	3rd
The Hippo pathway: adhesions-to-nucleus signaling and EMT control		
**YAP1**	**Transcriptional co-activator/co-repressor.** A major effector of the Hippo signaling. Plays a key role in the control of cell proliferation in response to cell contact. Regulates genes important for cell proliferation, cell death, and cell migration. Promotes cell growth, anchorage-independent growth, and epithelial mesenchymal transition (EMT).	2nd
**TAZ**	**Transcriptional co-activator/co-repressor.** A key effector of the Hippo signaling. Regulates the nuclear accumulation of SMADs and has a key role in coupling them to the transcriptional machinery. Regulates embryonic stem-cell self-renewal, promotes cell proliferation, and epithelial-mesenchymal transition (EMT).	2nd
Proto-oncogene signaling		
**MAFB**	**AP-1 transcriptional factor.** Maf proteins are bZIP transcription factors of the AP-1 superfamily. MAFB is involved in cell differentiation and hindbrain, otic, and lens development. Promotes synaptogenesis. A powerful regulator of cell-type specific post-synaptic density (PSD) development. Plays an essential role in central respiratory control.	2nd
**JUN**	**AP-1 transcriptional factor.** Regulates gene expression in response to a variety of stimuli, including cytokines, growth factors, stress, and bacterial and viral infections. Controls a number of cellular processes, including differentiation, proliferation, and apoptosis. Over-expressed in aggressive sarcomas. Promotes migration and invasiveness in breast and other cancers.	3rd
**FOS**	**AP-1 transcriptional factor.** Nuclear phosphoprotein that complex with the JUN/AP-1 transcription factor. On TGF-beta activation, forms a multimeric SMAD3/SMAD4/JUN/FOS complex at the AP1/SMAD-binding site to regulate TGF-beta-mediated signaling. It is thought to have an important role in cell proliferation and differentiation.	3rd

**ETS1**	**Transcriptional factor.** Directly controls the expression of cytokine and chemokine genes in a wide variety of different cellular contexts. May control the differentiation, survival and proliferation of lymphoid cells. May also regulate angiogenesis through regulation of expression of genes controlling endothelial cell migration and invasion.	3rd
TGF-beta signaling		
**TGF-beta**	**Transforming growth factor-beta.** Multifunctional protein that controls proliferation, differentiation and other functions in many cell types. Many cells synthesize TGF-beta and have specific receptors for it. Positively and negatively regulates other growth factors. A potent regulator of cell migration, differentiation, and proliferation. Promotes epithelial-mesenchymal transition (EMT).	1st
**MAP3K7**	**TGF-beta activated kinase 1 (TAK1).** Ser/Thr protein kinase. Acts as an essential component of the MAP kinase signal transduction pathway. Plays an important role in the cascades of cellular responses evoked by changes in the environment. Mediates signal transduction of TRAF6, various cytokines including IL-1, TGF-beta, and TGF-beta-related factors like BMP2 and BMP4. Acts as an upstream activator of the MKK/JNK signaling and the p38 MAPK signaling through the phosphorylation and activation of several MAP kinase kinases. These MAP2Ks in turn activate p38 MAPKs, c-jun N-terminal kinases (JNKs) and I-kappa-B kinase complex (IKK). Both p38 MAPK and JNK pathways control the transcription factors activator protein-1 (AP-1), while nuclear factor-kappa B (NF-kB) is activated by IKK.	2nd
**SMAD7**	**Transcriptional factor.** SMADs are transcription factors that mediate the signaling of TGF-beta-related factors. SMAD7 is a negative effector of TGF-beta signaling (an inhibitory SMAD). Implicated in both promotion and inhibition of tumorigenesis. May play an integral role in maintaining cell-cell adhesions through direct regulation of beta-catenin phosphorylation and stabilization of E-cadherin/catenin complexes.	3rd
Inflammation and immune responses; NF-kB signaling		
**IRF3**	**Interferon regulatory factor 3.** A key transcriptional regulator of type I interferon (IFN)-dependent immune response, which plays a critical role in the innate immune response against DNA and RNA viruses. Found in an inactive form in the cytoplasm of uninfected cells and following viral infection, double-stranded RNA (dsRNA), or toll-like receptor (TLR) signaling, is phosphorylated by IKBKE and TBK1 kinases. This induces a conformational change, leading to its dimerization and nuclear localization and association with CREB binding protein (CREBBP/CBP) to form dsRNA-activated factor 1 (DRAF1), a complex that activates the transcription of the type I IFN and IFN-stimulated genes.	2nd
**IKBKE**	**Inducible I kappa-B kinase epsilon.** Serine/threonine kinase that plays an essential role in regulating inflammatory responses to viral infection, through the activation of the type I IFN, NF-kB and STAT signaling. Also involved in TNFA and inflammatory cytokines, like IL-1, signaling. Phosphorylates interferon regulatory factors (IRFs), IRF3 and IRF7, as well as DDX3X. This activity allows subsequent homodimerization and nuclear translocation of the IRF3 leading to transcriptional activation of pro-inflammatory and antiviral genes including IFNB. Activated by polyubiquitination in response to TNFA and interleukin-1. Regulates the NF-kB signaling pathway through the phosphorylation of CYLD. Phosphorylates inhibitors of NF-kB thus leading to the dissociation of the inhibitor/NF-kB complex and ultimately the degradation of the inhibitor. Protects cells against DNA damage-induced cell death. Also plays an important role in energy balance regulation by sustaining a state of chronic, low-grade inflammation in obesity, which leads to a negative impact on insulin sensitivity. Phosphorylates AKT1.	3rd
**NFKBIA**	**NF-kappa-B inhibitor alpha.** Inhibits the activity of dimeric NF-kB/REL complexes by trapping REL dimers in the cytoplasm through masking of their nuclear localization signals. On cellular stimulation by immune and pro-inflammatory responses, becomes phosphorylated promoting ubiquitination and degradation, enabling the dimeric RELA to translocate to the nucleus and activate transcription.	3rd
